# Assessment of movement disorders using wearable sensors during upper limb tasks: A scoping review

**DOI:** 10.3389/frobt.2022.1068413

**Published:** 2023-01-09

**Authors:** Inti Vanmechelen, Helga Haberfehlner, Joni De Vleeschhauwer, Ellen Van Wonterghem, Hilde Feys, Kaat Desloovere, Jean-Marie Aerts, Elegast Monbaliu

**Affiliations:** ^1^ Research Group for Neurorehabilitation (eNRGy), KU Leuven Bruges, Department of Rehabilitation Sciences, Bruges, Belgium; ^2^ Amsterdam Movement Sciences, Amsterdam UMC, Department of Rehabilitation Medicine, Amsterdam, Netherlands; ^3^ Research Group for Neurorehabilitation (eNRGy), KU Leuven, Department of Rehabilitation Sciences, Leuven, Belgium; ^4^ Research Group for Neurorehabilitation (eNRGy), KU Leuven, Department of Rehabilitation Sciences, Pellenberg, Belgium; ^5^ Division of Animal and Human Health Engineering, KU Leuven, Department of Biosystems, Measure, Model and Manage Bioresponses (M3-BIORES), Leuven, Belgium

**Keywords:** inertial measurement unit (IMU), upper extremity, Parkinson’s disease, tremor, stroke, dystonia, ataxia, huntington’s disease

## Abstract

**Background:** Studies aiming to objectively quantify movement disorders during upper limb tasks using wearable sensors have recently increased, but there is a wide variety in described measurement and analyzing methods, hampering standardization of methods in research and clinics. Therefore, the primary objective of this review was to provide an overview of sensor set-up and type, included tasks, sensor features and methods used to quantify movement disorders during upper limb tasks in multiple pathological populations. The secondary objective was to identify the most sensitive sensor features for the detection and quantification of movement disorders on the one hand and to describe the clinical application of the proposed methods on the other hand.

**Methods:** A literature search using Scopus, Web of Science, and PubMed was performed. Articles needed to meet following criteria: 1) participants were adults/children with a neurological disease, 2) (at least) one sensor was placed on the upper limb for evaluation of movement disorders during upper limb tasks, 3) comparisons between: groups with/without movement disorders, sensor features before/after intervention, or sensor features with a clinical scale for assessment of the movement disorder. 4) Outcome measures included sensor features from acceleration/angular velocity signals.

**Results:** A total of 101 articles were included, of which 56 researched Parkinson’s Disease. Wrist(s), hand(s) and index finger(s) were the most popular sensor locations. Most frequent tasks were: finger tapping, wrist pro/supination, keeping the arms extended in front of the body and finger-to-nose. Most frequently calculated sensor features were mean, standard deviation, root-mean-square, ranges, skewness, kurtosis/entropy of acceleration and/or angular velocity, in combination with dominant frequencies/power of acceleration signals. Examples of clinical applications were automatization of a clinical scale or discrimination between a patient/control group or different patient groups.

**Conclusion:** Current overview can support clinicians and researchers in selecting the most sensitive pathology-dependent sensor features and methodologies for detection and quantification of upper limb movement disorders and objective evaluations of treatment effects. Insights from Parkinson’s Disease studies can accelerate the development of wearable sensors protocols in the remaining pathologies, provided that there is sufficient attention for the standardisation of protocols, tasks, feasibility and data analysis methods.

## Introduction

The execution of upper limb tasks requires fine-tuned coordination of multiple upper limb joints, which is often disturbed in individuals with movement disorders (Levin, 1996; Beer et al., 2000; Kukke et al., 2016). Movement disorders can be defined as “a neurological syndrome in which there is either an excess of movement or a paucity of voluntary and automatic movements” and are the consequence of lesions in the basal ganglia, cerebellum or thalamus brain regions. They are present in a variety of neurological diseases and can occur in every phase of the life cycle (Jankovic and Jankovic, 2021). Prevalence of movement disorders increases with age, up to 28% in a general population over 50 years old and 50% for individuals over 80 years old (Wenning et al., 2005). In several neurologic diseases, movement disorders belong to the main symptom of the disease. In childhood, neurologic movement disorders are most often associated with a diagnosis of dyskinetic cerebral palsy (CP) or with primary dystonias (i.e., inherited or idiopathic dystonias) with a prevalence of 25–50/100,000 and 15–30/100,000, respectively (Nutt et al., 1988; Phukan et al., 2011; Monbaliu et al., 2017). In individuals over the age of 50 years, the prevalence of primary dystonia increases to 732/100,000 (Müller et al., 2002). In the elderly, the most prevalent condition causing movement disorders is Parkinson’s disease (PD), reporting a prevalence of one to two per 1,000 adults (Tysnes and Storstein, 2017).

Movement disorders lead to slower movement execution, increased movement variability and a decrease in functionality (van den Noort et al., 2017; Newman et al., 2017; Sanger, 2006; Lee et al., 2015a; Zhang et al., 2012). Both in early-onset and late-onset movement disorders, accurate evaluation is indispensable for the follow-up of the disease course–especially in progressive movement disorders–and to evaluate and optimize the effect of treatment strategies. Currently, the effect of an intervention program on upper limb function or the presence and/or severity of movement disorders is mostly evaluated using clinical assessment scales such as functional scales and movement disorder severity scales (Jackman et al., 2016; Umar et al., 2018; Cohen et al., 2021). The Unified Parkinson’s Disease Rating Scale (UPDRS), the Movement Disorders Society revised version of this scale (MDS-UPDRS) and the Hoehn and Yahr scales are currently the most often used assessment scales in PD, whereas the Essential Tremor Rating Assessment Scale is used to rate the severity of essential tremor during nine functional tasks (Movement Disorder Society Task Force on Rating Scales for Parkinson’s Disease, 2003; Goetz et al., 2008; Hoehn and Yahr, 1967; Elble et al., 2012). To evaluate the severity of ataxia, the Scale for the Assessment and Rating of Ataxia (SARA) is most often applied (Schmitz-Hübschdu Montcel et al., 2006). In stroke, the Wolf Motor Function Test (WMFT) and Fugl-Meyer Assessment (FMA) are mainly used to evaluate motor function post-stroke (Movement Disorder Society Task Force on Rating Scales for Parkinson’s Disease, 2003; Hoehn and Yahr, 1967; Fugl-Meyer et al., 1975; Wolf et al., 2001; Gladstone et al., 2002). The Action Research Arm Test (ARAT), Box and Block test, Nine Hole Peg Test and Jebsen-Taylor Test evaluate hand function in multiple pathologies, amongst other multiple sclerosis (MS) and stroke, whereas the Monkey Box test was recently developed to evaluate bilateral motor function in Huntington’s Disease (HD) (Platz et al., 2005; Bennasar et al., 2018; Repnik et al., 2018). For children with CP, the Melbourne Assessment is a validated measure for upper limb activity (Gilmore et al., 2010; Spirtos et al., 2011). Apart from upper limb activity evaluation scales, the severity of movement disorders such as dystonia can be evaluated with the Burke–Fahn–Marsden Dystonia Rating Scale (BFMDRS) or the Dyskinesia Impairment Scale (DIS) in children and adolescents with dyskinetic CP (Burke et al., 1985; Monbaliu et al., 2012).

A common drawback of all abovementioned activity and movement disorder severity assessment scales is that they have to be evaluated by clinicians through the use of standardized guidelines or definitions with respect to task execution or presence/severity of the movement disorder. This clinical judgement induces subjectivity, as not all clinicians may interpret a definition or guideline in exactly the same manner. Moreover, the attribution of scores by a clinician based on video recordings is time-consuming, especially if frequent monitoring is required to evaluate disease progression or the effect of an intervention.

In an effort to reduce the subjective aspect in the evaluation of movement disorders, motion analysis has been widely introduced as an alternative to objectify movement disorders, as well as to evaluate the effect of treatment interventions in PD (Agostino et al., 2003; Pang et al., 2020), CP (Kreulen et al., 2006; Butler and Rose, 2012; Simon-Martinez et al., 2020) and stroke (Lang et al., 2009; Alt Murphy et al., 2018; Cuesta-GómezCarratala-Tejada et al., 2019). While three-dimensional motion analysis is the gold standard in movement analysis, it requires a specially equipped expensive laboratory whereby patients need to visit the hospital or study center for study participation or assessment of rehabilitation.

With both the time-consuming aspect of clinical scoring and the location-restricted aspect of three-dimensional motion analysis as main drivers, multiple studies have recently attempted to automate clinical scales with the use of wearable sensors or inertial measurement units (IMUs). These devices are attractive because of their ease-of-use and portability, omitting the necessity for a standardized laboratory which is in particular relevant for long-time follow-up or home-based measures for less mobile patients. IMUs measure linear acceleration and angular velocity of the segment they are placed on, whereas accelerometers measure only acceleration and gyroscopes measure only angular velocity. Specific features derived from acceleration and angular velocity measures can be used to characterize (pathological) movement patterns during multiple tasks or daily life activities. The use of wearable sensors for objective assessment has been previously discussed in PD (Maetzler et al., 2013), but this overview focused on all symptoms of PD, consequently providing very little information on specific upper limb tasks. Similarly, Tortelli and others discussed the use of portable digital sensors in HD, whereby the focus was mostly on the assessment of activity and gait (Tortelli et al., 2021). In dyskinetic CP, a recent review discussed instrumented measures for the assessment of dyskinetic CP symptoms, but this scope was not limited to IMUs and therefore less detailed on the topic (Haberfehlner et al., 2020). While these previous reviews provide much needed insights in the domain of each pathology, an overarching view of sensor protocols and features for the assessment of movement disorders during upper limb tasks could enhance standardisation of data collection. Such standardisation facilitates multi-centre studies and international collaborations and comparison between characteristics of movement disorders between diseases. Therefore, the primary objective of this review was to provide an overview of sensor set-up and type, included tasks, sensor features and methods that are used to evaluate movement disorders during upper limb tasks in multiple pathological populations. The secondary objective was to identify the most sensitive sensor features for the detection and quantification of movement disorders on the one hand and to describe the clinical application of the proposed methods on the other hand.

## Methods

### Search strategy

The full literature search was conducted following the Preferred Reporting Items for Systematic Reviews and Meta-Analyses (PRISMA) guidelines (Page et al., 2021). A literature search using three different databases was performed: Scopus, Web of Science, and PubMed until July 2022. Following terms were used in “all fields”:

#1: sensor OR inertial measurement unit; #2: arm OR upper limb; #3: movement disorder.

Subsequently, all three databases were searched for #1 AND #2 AND #3.

### Article screening

**FIGURE 1:**
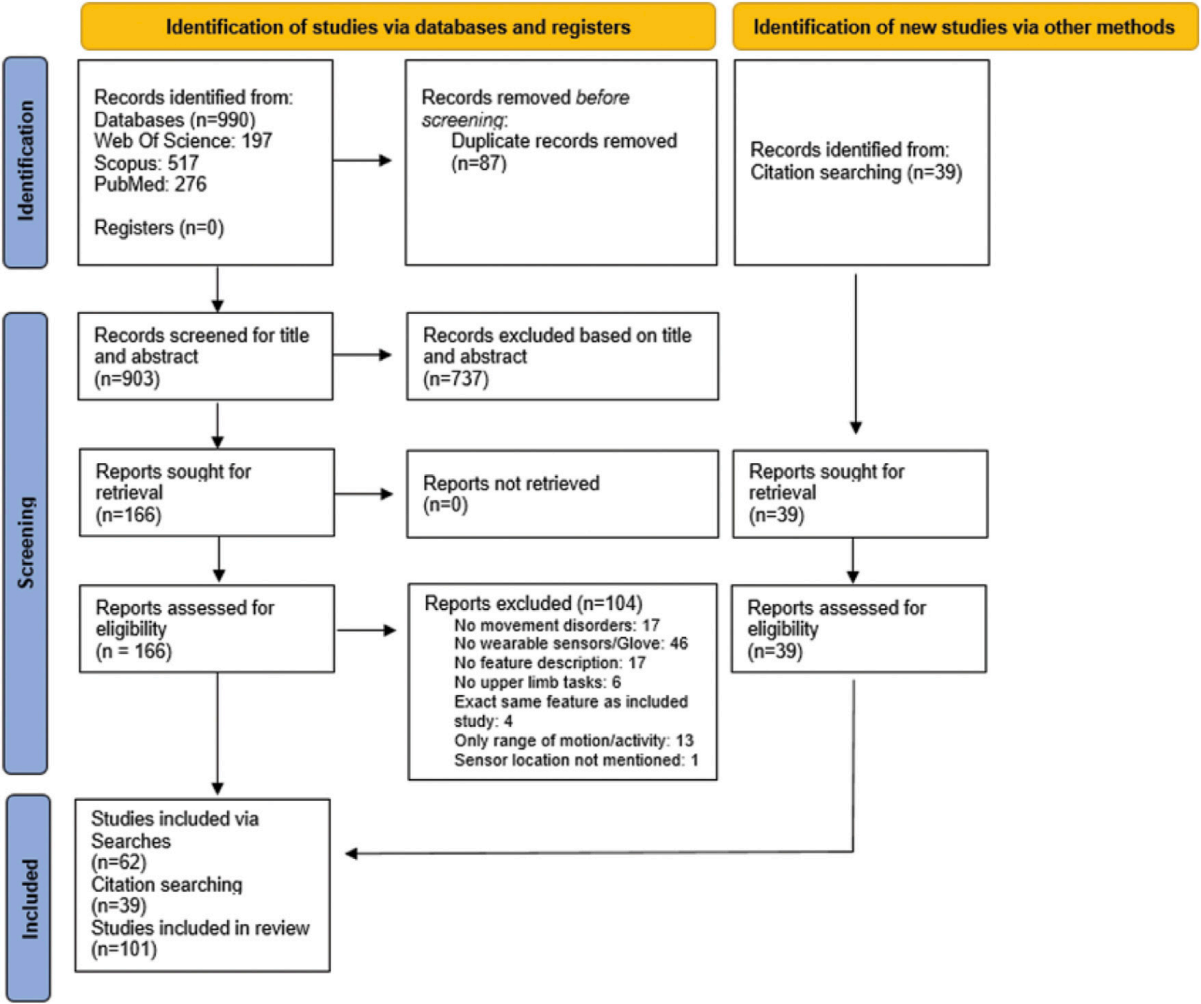
Flowchart of article selection.

Articles (n = 990) retrieved from the literature search were extracted. An overview of the articles retained at each stage of the screening process can be found in the PRISMA flow diagram presented in Figure 1 (Page et al., 2021). Any duplicated articles, retrieved by more than one database, were removed by de-duplication based on congruity in authors, title, and year of publication.

Unique articles (n = 903) were screened for inclusion by a researcher with experience in the field of upper extremity sensor measurements according to the criteria below in two consecutive stages: 1) title-abstract; and 2) full-text screening.

Articles were screened for inclusion along a set of pre-defined eligibility criteria for 1) the title-abstract and 2) the full-text screening stages. These criteria were designed in line with the PICO/PECO framework (Morgan et al., 2018), which clarifies the review objectives and inclusion criteria across four domains: (P) it was required that the participants were adults or children with a neurological disease subsequently leading to a movement disorder in (but not limited to) the upper limb. (I/E) a minimum of one wearable sensor was placed on the upper limb for the evaluation of movement disorders during the execution of an upper limb task. (C) Multiple comparisons were possible: 1) a group with movement disorders compared with a healthy group, 2) comparison of sensor features before and after an intervention or 3) comparison of sensor features with scores of a clinical scale. (O) Outcome measures needed to include sensor features derived from acceleration or angular velocity signals. Studies from the same authors who mentioned the exact same features in the same population as a study that was already included were excluded. Additionally, to meet the inclusion criteria, articles were required to be original research containing empirical data. Finally, only articles published after the year 2000 were included.

### Data extraction

Relevant information from each included article was extracted in a custom-made Excel based (Microsoft Office, Microsoft, Redmond, WA, United States) data extraction form. Information regarding goal population, sensor type, number of sensors, location of sensor(s), included tasks, sensor features and statistical method was obtained to address objective 1. To address objective 2, the sensitivity and/or responsiveness of the sensor features were extracted for the articles that provided the contribution of individual sensor features. Finally, the clinical application of the proposed method was extracted.

## Results

### General information

From the 166 full-text articles screened for eligibility, 62 were finally included. Additionally, 39 articles were included from citations of screened articles. The full-text articles that were screened but excluded and the reasons for exclusion can be found in Supplementary Table S1.

**FIGURE 2:**
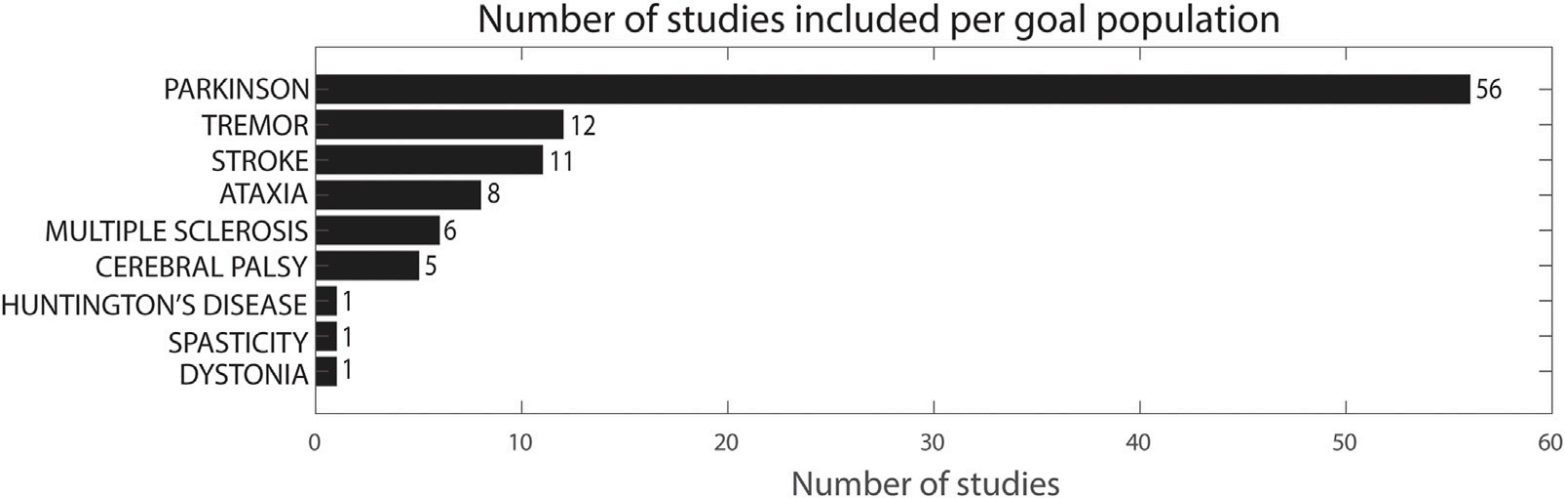
Number of studies included per goal population.

Of the included studies, 56 included adults with PD, of which 46 assessed one or multiple symptoms of PD and 10 studies specifically focused on Parkinsonian tremor (Makabe and Sakamoto, 2000; Rahimi et al., 2015; Thanawattano et al., 2015; Rigas et al., 2016; Bravo, 2017; Delrobaei et al., 2018; Lukšys et al., 2018; Zhu and Miller, 2020; Schaefer et al., 2021; Chan et al., 2022). Twelve studies included patients with essential tremor (Heldman et al., 2011b; Šprdlík et al., 2011; Gallego et al., 2012; Budini et al., 2014; Heo et al., 2015; Samotus et al., 2016; Chan et al., 2018; López-Blanco et al., 2018; Benito-LeónSerrano et al., 2019; Kwon et al., 2020; McGurrin et al., 2021; Ali et al., 2022) and 11 included adults post-stroke (Kamper et al., 2002; Knorr, 2005; Hester, 2006; Thies et al., 2009; Parnandi et al., 2010; Patel et al., 2010; Del Din et al., 2011; Zhang et al., 2012; Otten et al., 2015; van Meulen et al., 2015; Repnik et al., 2018), whereas six included adults with MS (Ketteringham, 2011; Carpinella et al., 2014; Carpinella et al., 2015; Teufl et al., 2017; Western et al., 2019; Teufl et al., 2021). One study included adults with HD and eight studies included children or adults with ataxia (Bennasar et al., 2018; Martinez-Manzanera et al., 2018; Krishna et al., 2019; Kashyap et al., 2020; Nguyen et al., 2020; Tran et al., 2020; Dominguez-Vega et al., 2021; Oubre et al., 2021; Gupta, 2022). Five studies included children with CP while two studies included children with dystonia and spasticity, respectively (Newman et al., 2017; Sanger, 2006; Strohrmann et al., 2013; Kim et al., 2018; den Hartogvan der Krogt et al., 2022; Legros et al., 2004; Bai et al., 2021) (Figure 2).

### Type, number and location of sensors

**FIGURE 3:**
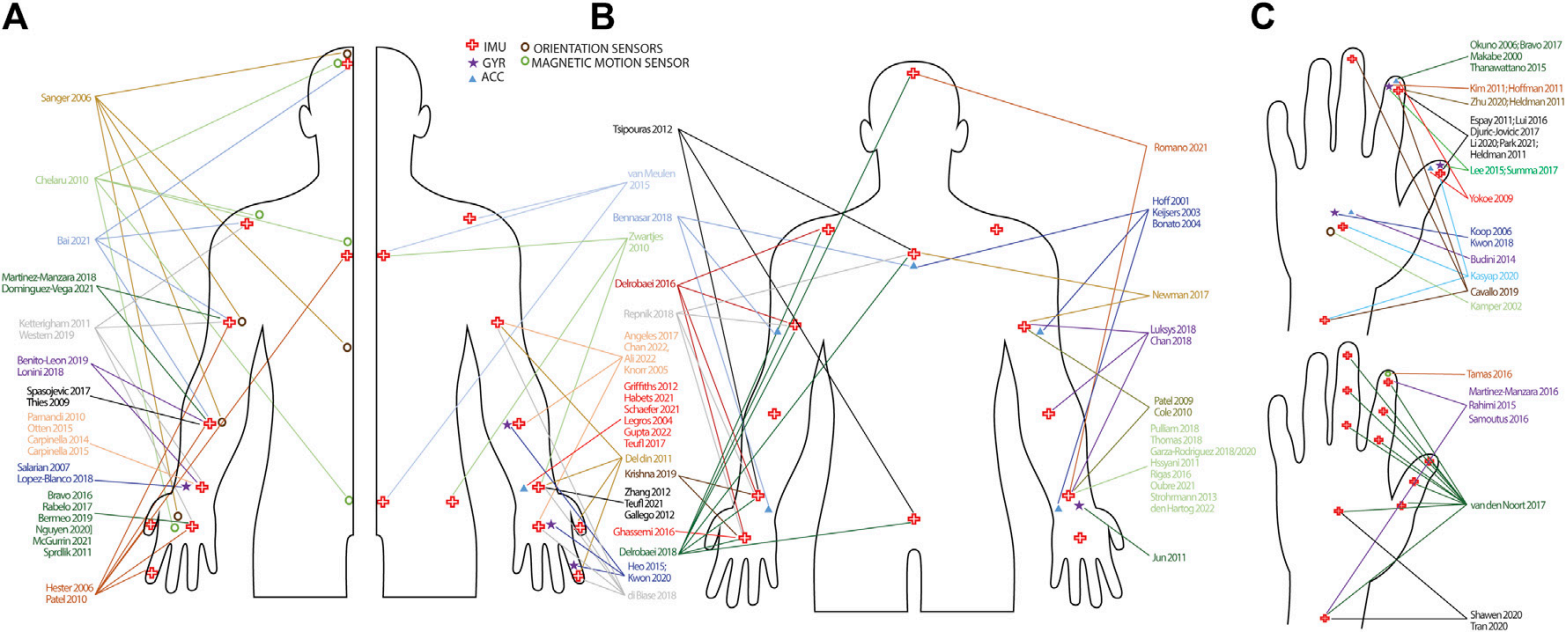
Infographic of sensor types and set-up. **(A)**: Studies including a unilateral sensor-set-up. Studies are randomly split in left and right side of the body to improve interpretation. **(B)**: Studies including a bilateral sensor set-up. Arrows have not been drawn to the contralateral side of the body to improve interpretation. **(C)**: Sensor placement on the hand and wrist. Hands are randomly split to improve interpretation. Sensor placements on the lower limb are not presented, but can be found in Supplementary Table S1. IMU = inertial measurement unit; GYR = gyroscope; ACC = accelerometer.

Figure 3 and Supplementary Table S2 yield an overview of type and number of sensors used, and their respective location for all included studies. From the 101 identified studies, 24 studies used an accelerometer (Makabe and Sakamoto, 2000; Hoff et al., 2001; Keijsers et al., 2003; Bonato et al., 2004; Legros et al., 2004; Knorr, 2005; Hester, 2006; Okuno et al., 2006; Patel et al., 2009; Yokoe et al., 2009; Cole et al., 2010; Patel et al., 2010; Del Din et al., 2011; Griffiths et al., 2012; Budini et al., 2014; Rahimi et al., 2015; Samotus et al., 2016; Bravo, 2017; Teufl et al., 2017; Bennasar et al., 2018; Kim et al., 2018; Habets et al., 2021; Schaefer et al., 2021; Gupta, 2022), 13 studies measured motion with a gyroscope or angular sensor (Koop et al., 2006; Salarian et al., 2007; Espay et al., 2011; Hoffman and McNames, 2011; Jun et al., 2011; Kim et al., 2011; Lee et al., 2015b; Heo et al., 2015; Summa et al., 2017; Kwon et al., 2018; López-Blanco et al., 2018; Kwon et al., 2020), four studies collected motion data using an orientation sensor, motion sensor or magnetic motion tracker (Sanger, 2006; Chelaru et al., 2010; di Biase et al., 2018; Kamper et al., 2002), 58 studies used IMUs including an accelerometer and gyroscope and three studies included IMUs but only used the acceleration signal for further analysis (Chan et al., 2018; Benito-LeónSerrano et al., 2019; Ali et al., 2022), while one study used IMUs but only processed angular velocity signals (Thanawattano et al., 2015).

The number of sensors ranged from one to 17. Thirty-seven studies used only one sensor, either on the finger, hand, wrist or forearm (Makabe and Sakamoto, 2000; Kamper et al., 2002; Legros et al., 2004; Koop et al., 2006; Okuno et al., 2006; Salarian et al., 2007; Thies et al., 2009; Parnandi et al., 2010; Heldman et al., 2011b; Hoffman and McNames, 2011; Kim et al., 2011; Šprdlík et al., 2011; Gallego et al., 2012; Griffiths et al., 2012; Zhang et al., 2012; Budini et al., 2014; Carpinella et al., 2014; Carpinella et al., 2015; Otten et al., 2015; Thanawattano et al., 2015; Bravo, 2016; Rigas et al., 2016; Tamás et al., 2016; Bravo, 2017; Rabelo et al., 2017; Spasojević et al., 2017; Teufl et al., 2017; Kwon et al., 2018; López-Blanco et al., 2018; Bermeo, 2019; Nguyen et al., 2020; Zhu and Miller, 2020; Habets et al., 2021; McGurrin et al., 2021; Schaefer et al., 2021; Teufl et al., 2021; Gupta, 2022), while seven studies used two sensors bilaterally placed on the hand or wrist (Jun et al., 2011; Strohrmann et al., 2013; Ghassemi et al., 2016; Garza-RodríguezSanchez-Fernandez et al., 2018; Thomas et al., 2018; Garza-RodríguezSanchez-Fernandez et al., 2020; Oubre et al., 2021). Nine studies used two sensors of which the majority placed one on the thumb and one the index finger (Yokoe et al., 2009; Heldman et al., 2011a; Espay et al., 2011; Lee et al., 2015b; Djurić-JovičićPetrovic et al., 2016; Liu et al., 2016; Summa et al., 2017; Li et al., 2020; Park et al., 2021), while Martinez-Manzara et al. used one sensor on the hand and one on the finger (Martinez-Manzanera et al., 2016), Samotus et al. and Rahimi et al. put one on the wrist and one on the index finger (Rahimi et al., 2015; Samotus et al., 2016) and Shawen et al. put one on the hand and one on the wrist (Shawen et al., 2020). In the studies where three sensors were used, the most frequent locations for PD and tremor were hand, forearm and upper arm (Knorr, 2005; Angeles et al., 2017; Ali et al., 2022; Chan et al., 2022), index finger, hand, forearm (Heo et al., 2015; Kwon et al., 2020) or index finger, forearm and upper arm (Martinez-Manzanera et al., 2018; Dominguez-Vega et al., 2021). In children with CP, Newman et al. attached one sensor on the sternum and two on both upper arms (Newman et al., 2017) and in HD, Bennasar et al. placed one sensor on the sternum and two on the wrists (Bennasar et al., 2018). In stroke, Van Meulen et al. fixed one sensor on the wrist, sternum and sacrum (van Meulen et al., 2015). When four sensor were used, sensor placements were: three fingers and the wrist in PD (Cavallo et al., 2019), thumb, index finger, wrist and upper arm in stroke (Del Din et al., 2011), hand, forearm, upper arm and shoulder in spasticity (Bai et al., 2021), hands and forearms (Benito-LeónSerrano et al., 2019; Romano et al., 2021) and wrists, trunk and head in PD (Lonini et al., 2018). Four more studies also included lower limb sensors, where three placed sensors on both wrists and ankles and Zwartjes et al. included wrist, foot, thigh and sternum (Zwartjes et al., 2010; Pulliam et al., 2018; Hssayeni et al., 2021; den Hartogvan der Krogt et al., 2022). Four studies used five sensors on the upper limbs, with sensor placement on hand, wrist, upper arm, shoulder and sternum in MS (Ketteringham, 2011; Western et al., 2019), thumb, index finger, hand, forearm, upper arm and sternum for Di Biase et al. and hand forearm, upper arm, head and back for Sanger et al. (note that both studies are based on magnetic or orientation sensors) (Sanger, 2006; di Biase et al., 2018). Seven studies used six sensors, where the sensor placement was thumb, index finger, hand, forearm, upper arm and sternum (Hester, 2006; Patel et al., 2010). Two studies placed the sensors on hands, forearms and upper arms (Chan et al., 2018; Lukšys et al., 2018), while Krishna et al. used one sensor but subsequently fixed it on both hands, wrists and ankles, thus including six sensor signals in the analysis (Krishna et al., 2019). Cheralu et al. placed sensors on the hand, scapula, thorax, sacrum, head and shank, while Tsipouras et al. attached sensors bilaterally on ankles and wrists and one the waist and chest (Chelaru et al., 2010; Tsipouras et al., 2012). Repnik et al. used seven sensors on the hands, wrists, upper arms and sternum and Hof et al. and Keijsers et al. placed the sensors on the wrists, upper arms, trunk and upper legs (Hoff et al., 2001; Keijsers et al., 2003; Repnik et al., 2018). Five studies used eight sensors. Delrobaei et al. placed the sensors on the hands, wrists, upper arms, and shoulders and Bonato et al. on the forearms, upper arms, thighs, right shin and sternum (Bonato et al., 2004; Delrobaei et al., 2016). Patel et al. and Cole and others attached the sensors on the forearms, upper arms, shins and upper legs (Patel et al., 2009; Cole et al., 2010), whereas in ataxia, Kashyap and others placed sensors on the index finger, hand, wrist, foot, sternum, back and ankles (Kashyap et al., 2020). Finally, Van den Noort et al. used 11 sensors, all located on the hand and fingers (van den Noort et al., 2017) and Delrobaei and others used 17 sensors placed on the hands, wrists, upper arms, clavicle’s, sternum, head, pelvis, upper and lower legs and feet (Delrobaei et al., 2018). In one study, the number of sensors was not specified (Kim et al., 2018).

### Upper limb tasks

The upper limb tasks occurring in more than one study are listed in Table 1. Wrist pro/supination was included in 25 studies whereas finger tapping was included in 23 studies, with the majority being studies in PD patients. Keeping arms in front of the body was included in 23 studies in PD, tremor and MS, as well as finger-to-nose which was additionally included in one ataxia study. Drinking from a can/cup was included in 13 studies in PD, tremor, stroke and spasticity. Opening/closing of the hand was included in seven PD studies as well as writing/drawing which was used for PD and tremor patients. Eating was included in six PD studies as well as pouring water, which was used in PD, tremor and MS. Reaching/grasping to objects was included in five studies in stroke, ataxia and CP and teeth brushing and putting clothes on/off were both used four times in PD studies. In stroke, the Wolf Motor Function test or parts of this clinical scale were included four times and two PD, one ataxia and one CP study measured activities in an unrestricted home environment. Combing hair, typing and folding laundry were included in three PD studies and forwards and sideways reaching in one CP and two stroke studies. The box and block test was included in one PD and one CP study and tasks from the ARAT were additionally included in PD and one CP study. Finally, following tasks were included once: reaching sideways (Newman et al., 2017), the monkey box test (Bennasar et al., 2018), holding a weight with the wrist (Schaefer et al., 2021), wrist extension (Rabelo et al., 2017), wrist ab/adduction, flexion/extension, elbow flexion/extension and pro/supination (Chan et al., 2022), and following a bent wire shape with a wand loop (Budini et al., 2014). One study included wrist supination/flexion, hand behind back and wrist flexion/pronation (Zhang et al., 2012). In CP, one study included outwards reaching (Sanger, 2006), one included the drinking test, the bean bag test and the nine hole peg test (Bai et al., 2021) while Strohrmann et al. included turn around cards, pick up small objects, stack dominos, open and close and bottle, use a key, and the nine-hole peg test (Strohrmann et al., 2013). Kim et al. included the Jebsen Taylor Hand Function Test, the Quality of Upper Extremity Skills Test and the abovementioned Box and Blocks Test (Kim et al., 2018).

**TABLE 1:** Tasks included in more than one study, the number of studies and the goal population with associated references.

Task	^#^ Of studies	Goal population
Wrist pro/supination	25	PD van den Noort et al. (2017); Koop et al. (2006); Salarian et al. (2007); Patel et al. (2009); Espay et al. (2011); Jun et al. (2011); Delrobaei et al. (2016); Tamás et al. (2016); Spasojević et al. (2017); Summa et al. (2017); Angeles et al. (2017); Lonini et al. (2018); Thomas et al. (2018); Kwon et al. (2018); di Biase et al. (2018); Garza-RodríguezSanchez-Fernandez et al. (2018); Garza-RodríguezSanchez-Fernandez et al. (2020); Cavallo et al. (2019); Shawen et al. (2020); Park et al. (2021); Heldman et al. (2011a); Tremor McGurrin et al. (2021); Ataxia Krishna et al. (2019); Tran et al. (2020); Dominguez-Vega et al. (2021)
Finger tapping	23	PD van den Noort et al. (2017); Okuno et al. (2006); Patel et al. (2009); Yokoe et al. (2009); Espay et al. (2011); Kim et al. (2011); Hoffman and McNames, (2011); Lee et al. (2015b); Martinez-Manzanera et al. (2016); Tamás et al. (2016); Liu et al. (2016); Djurić-JovičićPetrovic et al. (2016); Spasojević et al. (2017); Summa et al. (2017); Kwon et al. (2018); di Biase et al. (2018); Cavallo et al. (2019); Li et al. (2020); Park et al. (2021); Heldman et al. (2011a); Ataxia Kashyap et al. (2020); Nguyen et al. (2020); Tran et al. (2020)
Keeping arms in front of the body	23	PD Salarian et al. (2007); Budini et al. (2014); Heo et al. (2015); Rahimi et al. (2015); Thanawattano et al. (2015); Ghassemi et al. (2016); Rigas et al. (2016); Bravo, (2017); Delrobaei et al. (2018); Lukšys et al. (2018); Benito-LeónSerrano et al. (2019); Zhu and Miller, (2020); Tremor Heldman et al. (2011b); Šprdlík et al. (2011); Gallego et al. (2012); Samotus et al. (2016); Chan et al. (2018); López-Blanco et al. (2018); Kwon et al, (2020); MS Ketteringham, (2011); Teufl et al. (2017); Western et al. (2019); Teufl et al. (2021)
Finger to nose	23	CP Sanger, (2006); PD Patel et al. (2009); Thanawattano et al. (2015); Bravo, (2017); Lonini et al. (2018); Lukšys et al. (2018); Shawen et al. (2020); Zhu and Miller (2020); Tremor Heldman et al. (2011b); Gallego et al. (2012); Budini et al. (2014); López-Blanco et al. (2018); MS Ketteringham, (2011); Carpinella et al. (2015); Teufl et al. (2017); Krishna et al. (2019); Western et al. (2019); Tran et al. (2020); Teufl et al. (2021); Ataxia Martinez-Manzanera et al. (2018); Kashyap et al. (2020); Dominguez-Vega et al. (2021); Oubre et al. (2021)
Drinking from a can/cup	13	PD Hoff et al. (2001); Keijsers et al. (2003); Salarian et al. (2007); Zwartjes et al. (2010); Tsipouras et al. (2012); Lonini et al. (2018); Shawen et al. (2020); Hssayeni et al. (2021); Tremor Heldman et al. (2011b); Chan et al. (2018); Stroke Thies et al. (2009); Patel et al. (2010); Spasticity Bai et al. (2021)
Opening/closing hand	7	PD van den Noort et al. (2017); Espay et al. (2011); Tamás et al. (2016); Djurić-JovičićPetrovic et al. (2016); Cavallo et al. (2019); Park et al. (2021); Heldman et al. (2011a)
Writing/drawing	7	PD Salarian et al. (2007); Lonini et al. (2018); Pulliam et al. (2018); Shawen et al. (2020); Tremor Heldman et al. (2011b); McGurrin et al. (2021); Ali et al. (2022)
Eating	6	PD Keijsers et al. (2003); Salarian et al. (2007); Bravo, (2016); Pulliam et al. (2018); Bermeo, (2019); Hssayeni et al. (2021)
Pouring water	6	PD Lonini et al. (2018); Shawen et al. (2020); Tremor Heldman et al. (2011b); López-Blanco et al. (2018); MS Teufl et al. (2017); Teufl et al. (2021)
Reaching/grasping objects	5	Stroke Thies et al. (2009); van Meulen et al. (2015); Ataxia Teufl et al. (2017); Teufl et al. (2021); CP Strohrmann et al. (2013)
Teeth brushing	4	PD Salarian et al. (2007); Bravo, (2016); Pulliam et al. (2018); Bermeo, (2019)
Putting clothes on/off	4	PD Hoff et al. (2001); Keijsers et al. (2003); Pulliam et al. (2018); Hssayeni et al. (2021)
Wolf Motor Function test	4	Stroke Hester, (2006); Parnandi et al. (2010); Patel et al. (2010); Del Din et al. (2011)
Unrestricted home activities	3	PD Griffiths et al. (2012); Romano et al. (2021); Ataxia Gupta, (2022); CP den Hartogvan der Krogt et al. (2022)
Combing hair	3	PD Salarian et al. (2007); Pulliam et al. (2018); Hssayeni et al. (2021)
Typing	3	PD Lonini et al. (2018); Pulliam et al. (2018); Shawen et al. (2020)
Folding laundry	3	PD Lonini et al. (2018); Pulliam et al. (2018); Shawen et al. (2020)
Forwards and sideways reaching	3	CP Newman et al. (2017); Stroke Kamper et al. (2002); Knorr (2005)
ARAT	2	PD Romano et al. (2021); CP Kim et al. (2018)
Box and block test	2	Stroke Repnik et al. (2018); MS Carpinella et al. (2014)

### Sensor features

Table 2 provides an overview of the calculated sensor features in the time-and frequency domain, as well as a formula or feature description when given in the original study. Supplementary Table S3 presents the sensor features grouped per pathology. As an easy and straightforward feature, execution time was often calculated for the upper limb tasks for stroke (Thies et al., 2009; Zhang et al., 2012; Repnik et al., 2018), MS (Carpinella et al., 2014), PD (van den Noort et al., 2017; Bonato et al., 2004; Yokoe et al., 2009; Djurić-JovičićPetrovic et al., 2016; Rabelo et al., 2017; di Biase et al., 2018), tremor (Tran et al., 2020; Ali et al., 2022), CP (Strohrmann et al., 2013) and ataxia (Dominguez-Vega et al., 2021; Gupta, 2022). The frequency of movements was popular in multiple studies in PD, mostly in repetitive tasks such as finger tapping and pro/supination (Lee et al., 2015b; Cavallo et al., 2019).

**TABLE 2:** Calculated sensor features in time and frequency-domain. STD = standard deviation; max = maximal; RMS = root-mean-square; VAR = variance; IQR = inter-quartile range. PSD = power spectral density.

Time-domain	Formula/Feature description	Goal population
Execution time	$T_{m_{i}}=T_{t_{i}}-T_{0_{i}}$ Repnik et al. (2018); Inter-tap-interval (ITI) Tran et al. (2020); mean, standard deviation, minimum, maximum, range, interquartile range, median, and 10th and 90th percentiles of time Oubre et al. (2021); duration of sub movements Gupta, (2022)	Stroke Thies et al. (2009); Zhang et al. (2012); Repnik et al. (2018), PD van den Noort et al. (2017); Bonato et al. (2004); Yokoe et al. (2009); Djurić-JovičićPetrovic et al. (2016); Rabelo et al. (2017); di Biase et al. (2018), CP Strohrmann et al. (2013); Newman et al. (2017), tremor Tran et al. (2020); Ali et al. (2022), MS (Carpinella et al. (2014); Carpinella et al. (2015), ataxia Dominguez-Vega et al. (2021); Gupta, (2022)
Movement Frequency	Number of rotations/movements Lee et al. (2015b)	PD Lee et al. (2015b); Cavallo et al. (2019)
Mean acceleration and angular velocity	$m_{i}\left(k\right)=\frac{1}{2sf+1}\overset{w\left(k\right)+sf}{\underset{n=w\left(k\right)-sf}{\sum }}x_{i}\left(n\right)$ Tsipouras et al. (2012); Mean absolute value; Absolute and harmonic mean den Hartogvan der Krogt et al. (2022)	PD Tsipouras et al. (2012); Thomas et al. (2018); Shawen et al. (2020); Romano et al. (2021), stroke Parnandi et al. (2010), dyskinetic CP den Hartogvan der Krogt et al. (2022), ataxia Krishna et al. (2019)
STD acceleration and angular velocity	$s_{i}\left(k\right)=\sqrt{\frac{1}{2sf+1}\overset{w\left(k\right)+sf}{\underset{n=w\left(k\right)-sf}{\sum }}(x_{i}^{f}\left(n\right)-{\bar{x}}_{i}^{f}{)}^{2}²}$ Tsipouras et al. (2012); $\sigma \left(W\right)=\sqrt{\frac{1}{N}\overset{N}{\underset{i=1}{\sum }}(x_{i}-\mu \left(W\right){)}^{2}²},\;\mu \left(W\right)=mean$ Hssayeni et al. (2021)	PD Tsipouras et al. (2012); Thomas et al. (2018); Hssayeni et al. (2021)
MAX acceleration and angular velocity	Absolute max Rabelo et al. (2017)	PD Rabelo et al. (2017); Habets et al. (2021), dyskinetic CP den Hartogvan der Krogt et al. (2022)
Timing of MAX acceleration and angular velocity	Absolute max Rabelo et al. (2017)	PD Rabelo et al. (2017)
RMS acceleration and angular velocity		PD van den Noort et al. (2017); Shawen et al. (2020), dyskinetic CP den Hartogvan der Krogt et al. (2022), ataxia Krishna et al. (2019)
VAR acceleration and angular velocity		Stroke Parnandi et al. (2010); PD Shawen et al. (2020); Habets et al. (2021), ataxia Krishna et al. (2019)
Mean acceleration	Mean acceleration in 2min epoch Griffiths et al. (2012)	Stroke Hester, (2006); Patel et al. (2010); Del Din et al. (2011), PD Zwartjes et al. (2010); Griffiths et al. (2012); Ghassemi et al. (2016); Schaefer et al. (2021), HD Bennasar et al. (2018), CP Strohrmann et al. (2013), tremor Samotus et al. (2016), ataxia Gupta, (2022)
STD acceleration		PD Yokoe et al. (2009); Ghassemi et al. (2016); Habets et al. (2021) HD Bennasar et al. (2018), CP Strohrmann et al. (2013), tremor Budini et al. (2014), ataxia Gupta, (2022)
MAX acceleration	Max acceleration in 2min epoch Griffiths et al. (2012); Moments of jerk magnitude Habets et al. (2021)	PD Griffiths et al. (2012); Habets et al. (2021), CP Kim et al. (2018), ataxia Oubre et al. (2021)
Mean angular velocity	Hand mobility Salarian et al. (2007)	PD Salarian et al. (2007); Lee et al. (2015b); Djurić-JovičićPetrovic et al. (2016); Angeles et al. (2017); Garza-RodríguezSanchez-Fernandez et al. (2018); Cavallo et al. (2019); Li et al. (2020), ataxia Dominguez-Vega et al. (2021); Oubre et al. (2021)
STD angular velocity		PD Angeles et al. (2017), ataxia Oubre et al. (2021)
Median angular velocity		PD Garza-RodríguezSanchez-Fernandez et al. (2020)
Median acceleration		PD Habets et al. (2021)
MAX linear velocity	Average of maximum velocities Okuno et al. (2006)	PD Okuno et al. (2006); Yokoe et al. (2009), stroke Hester, (2006)
RMS acceleration		PD Patel et al. (2009); Ghassemi et al. (2016); Habets et al. (2021); stroke Knorr, (2005); Hester, (2006)
RMS angular velocity	Speed Tamás et al. (2016); Mean Intensity (MI) = RMS angular velocity López-Blanco et al. (2018)	PD Koop et al. (2006); Heldman et al. (2011a); Espay et al. (2011); Jun et al. (2011); Kim et al. (2011); Tamás et al. (2016); Kwon et al. (2018); Lukšys et al. (2018); Zhu and Miller, (2020); Park et al. (2021), tremor Heo et al. (2015); López-Blanco et al. (2018); Kwon et al. (2020)
Mean angular displacement		MS Western et al. (2019)
RMS angular displacement/movement amplitude	Amplitude Tamás et al. (2016)	PD Heldman et al. (2011a); Espay et al. (2011); Jun et al. (2011); Kim et al. (2011); Tamás et al. (2016); Delrobaei et al. (2018); Kwon et al. (2018); Cavallo et al. (2019); Chan et al. (2022), tremor Chan et al. (2018); Kwon et al. (2020)
Range angular displacement	Hand activity Salarian et al. (2007)	PD van den Noort et al. (2017); Salarian et al. (2007); Djurić-JovičićPetrovic et al. (2016); Garza-RodríguezSanchez-Fernandez et al. (2020); Cavallo et al. (2019); Romano et al. (2021)
IQR angular displacement		PD Garza-RodríguezSanchez-Fernandez et al. (2020)
Range acceleration		PD Patel et al. (2009); Yokoe et al. (2009); Habets et al. (2021), stroke Otten et al. (2015)
Range acceleration and angular velocity		PD (Rabelo et al. (2017); Lonini et al. (2018); Shawen et al. (2020)
Mean amplitude	Average amplitude Okuno et al. (2006)	PD Okuno et al. (2006); Lee et al. (2015b)
Range angular velocity	RAV = $\frac{1}{3}\underset{frontal,vertical,lateral}{\sum }range\left(\omega \right)$	PD Rabelo et al. (2017); di Biase et al. (2018), CP Strohrmann et al. (2013); Newman et al. (2017), ataxia Oubre et al. (2021)
IQR acceleration and angular velocity	Angular velocity Garza-RodríguezSanchez-Fernandez et al. (2020); angular acceleration Habets et al. (2021)	PD Garza-RodríguezSanchez-Fernandez et al. (2020); Habets et al. (2021)
Range of jerk and angular acceleration	${\left(Acc/Gyr\right)}_{\mathrm{ran}}=\max\left(\dot{x}\left(t\right)\right)-\min\left(\dot{x}\left(t\right)\right)\;t\in \left\{1,W_{n}\right\}$ Spasojević et al. (2017)	PD Spasojević et al. (2017)
Peak-to-peak angular velocity	Difference between the mean of the highest and lowest 10 samples in W Hssayeni et al. (2021)	PD Summa et al. (2017); di Biase et al. (2018); Hssayeni et al. (2021))
Magnitude angular velocity		PD Pulliam et al. (2018)
Max, STD, RMS and min/max peak height		HD Bennasar et al. (2018)
Rotational jerk index	${ղ}_{rot_{i}}=\log\sqrt{\frac{\left(T_{t_{i}-}T_{0_{i}}\right)5}{2\theta^{p}}\overset{T_{t_{i}}}{\underset{T_{0_{i}}}{\int }}∥\frac{d^{2}²\omega \left(t\right)}{{dt}^{2}²}{∥}^{2}dt}$ Repnik et al. (2018)	Stroke Repnik et al. (2018)
Segment velocity	Square root of sum of squares of jerk signals in three directions Keijsers et al. (2003)	PD Keijsers et al. (2003)
Kurtosis	SW=1N∑i=1N(xi−μ(W))4σW4 Hssayeni et al. (2021)	PD Parnandi et al. (2010) Ghassemi et al. (2016); Lonini et al. (2018); Shawen et al. (2020); Hssayeni et al. (2021), ataxia Gupta, (2022)
Skewness	SW=1N∑i=1N(xi−μW)3³σ(W)3³ Hssayeni et al. (2021	PD Parnandi et al. (2010); Ghassemi et al. (2016); Lonini et al. (2018); Thomas et al. (2018); Shawen et al. (2020); Hssayeni et al. (2021)
Sample entropy	$H_{i}\left(k\right)=-\frac{1}{2sf+1}\overset{n=w\left(k\right)+sf}{\underset{n=w\left(k\right)-sf}{\sum }}p\left(x_{i}^{f}\left(n\right)\right)logp\left(x_{i}^{f}\left(n\right)\right)$ Tsipouras et al. (2012)	PD Patel et al. (2009); Chelaru et al. (2010); Tsipouras et al. (2012); Ghassemi et al. (2016); Liu et al. (2016); Lonini et al. (2018); Shawen et al. (2020), HD Bennasar et al. (2018), stroke Patel et al. (2010), ataxia (Kashyap et al. (2020)
Approximate entropy	***H(W) = −∑p(Bi) ∗*** **log** ***p(Bi)*** Hester, (2006); Window length (m) = 2 and % STD (r) = 20% Thomas et al. (2018)	PD Bonato et al. (2004); Parnandi et al. (2010); Liu et al. (2016); Lukšys et al. (2018); Thomas et al. (2018), stroke Knorr, (2005); Hester, (2006)
Shannon entropy	Randomness in time domain: $H\left(W\right)=-\overset{200}{\underset{i=1}{\sum }}p\left(B_{i}\right)*\log p\left(B_{i}\right)$ Hssayeni et al. (2021)	PD Hssayeni et al. (2021), dyskinetic CP den Hartogvan der Krogt et al. (2022)
Permutation entropy	Assesses the complexity of the time series signal Bennasar et al. (2018)	HD Bennasar et al. (2018)
Fuzzy entropy		Ataxia Nguyen et al. (2020); Tran et al. (2020)
Gini index	Movement complexity in the time domain: $G\left(W\right)=1-\overset{200}{\underset{i=1}{\sum }}p(B_{i}{)}^{2}²$ Hssayeni et al. (2021)	PD Hssayeni et al. (2021), ataxia Nguyen et al. (2020)
Lyapunov exponent	Measures the level of chaos in a signal Bennasar et al. (2018)	HD Bennasar et al. (2018)
Recurrence rate (RR); Determinism	RR: Probability that any state will recur again; Determinism: Ratio of recurrence points	HD Bennasar et al. (2018)
Average Diagonal line	Average time that signal segments remain the same Bennasar et al. (2018)	HD Bennasar et al. (2018)
RMS of jerk		PD Parnandi et al. (2010), stroke Knorr (2005); Hester (2006)
Jerk metric	RM jerk normalized by peak velocity Hester (2006); Moments of jerk magnitude Lonini et al. (2018); Habets et al. (2021); Logarithm of mean jerk amplitude, normalized to mean absolute acceleration movement duration Carpinella et al. (2014); Normalised Jerk Index: NJI = $\frac{1}{v_{peak}\left(t_{1-t_{2}}\right)}\overset{t_{2}}{\underset{t_{1}}{\int }}\left|\frac{d^{2}v}{{dt}^{2}²}\right|dt$ Newman et al. (2017); Bai et al. (2021); Dimensionless Jerk Index (DLJ) Romano et al. (2021); Mean jerk Zhang et al. (2012)	Stroke Hester (2006); Zhang et al. (2012), PD (Lonini et al. (2018); Habets et al. (2021); Romano et al. (2021), MS Carpinella et al. (2014), CP Newman et al. (2017), dyskinetic CP Sanger (2006), spasticity Bai et al. (2021)
Smoothness	Difference between movement accelerometer readings and smoothed readings Otten et al. (2015); Number of movement units [BAI]; number of speed peaks (NSP) Kamper et al. (2002)	Stroke Otten et al. (2015), spasticity Bai et al. (2021)
Coefficient of variation	Coefficient of variation of amplitude, speed and frequency (Lee et al. (2015b); Djurić-JovičićPetrovic et al. (2016); STD of a 1-s sliding window of the RMS excursion angle divided by the mean and Coefficient of variation of acceleration and angular velocity Espay et al. (2011); Coefficient of variation of excursion angle (Heldman et al. (2011a); Espay et al. (2011); Tamás et al. (2016); Kwon et al., 2018); Coefficient of variation of angular velocity Kwon et al. (2018); Coefficient of variation of inter-tap -interval Tran et al. (2020)	PD Heldman et al. (2011a); Espay et al. (2011); Lee et al. (2015b); Djurić-JovičićPetrovic et al. (2016); Tamás et al. (2016); Kwon et al. (2018); Cavallo et al. (2019), tremor Tran et al. (2020)
Rhythm	STD of intervals a single finger tap movement in 60 s Okuno et al. (2006); Any sequence of regularly recurring events (Martinez-Manzanera et al. (2016)	PD Okuno et al. (2006); Martinez-Manzanera et al. (2016)
Variability	RMS error between the References trial and the warped trial Thies et al. (2009)	Stroke Thies et al. (2009)
Higuchu’s fractal dimension (HFD)	Geometrical structure of non-linear time series Newman et al. (2017)	CP Newman et al. (2017)
Correlation between axes	Mean, STD, skewness and kurtosis of signal derivative Shawen et al. (2020); Correlation between each two axes of accelerometer Bennasar et al. (2018)	PD Lonini et al. (2018); Shawen et al. (2020); Zhu and Miller, (2020); Habets et al. (2021), HD Bennasar et al. (2018), stroke Hester, (2006)
Peak of normalized cross-correlation from pairs of acceleration time series		PD Patel et al. (2009)
Lag of first peak in autocorrelation acceleration		PD Cole et al. (2010)
Path length	Path-length-ratio (PLR) = distance travelled by hand/straight-line distance Kamper et al. (2002); Length of 3D trajectories van Meulen et al. (2015); Index of curvature Sanger (2006); Mean and Standard deviation of Euclidian distance from the mean trajectory Dominguez-Vega et al. (2021); curved line similarity analysis, straight line similarity analysis Martinez-Manzanera et al. (2018)	Stroke Kamper et al. (2002); van Meulen et al. (2015), dyskinetic CP Sanger (2006), spasticity (Bai et al. (2021), ataxia (Dominguez-Vega et al. (2021)
Similarity of hand trajectories	$D_{i}=\sqrt{\frac{1}{{\bar{s}}_{t_{i}}-{\bar{s}}_{0_{i}}}\overset{{\bar{s}}_{t_{i}}}{\underset{{\bar{s}}_{0_{i}}}{\int }}∥p\left(\bar{s}\right)-p_{r}\left(\bar{s}\right){∥}^{2}²d\bar{s}}$ , ${\hat{v}}_{i}=\frac{1}{\pi }\sqrt{\frac{1}{{\bar{s}}_{t_{i}}-{\bar{s}}_{0_{i}}}\overset{{\bar{s}}_{t_{i}}}{\underset{{\bar{s}}_{0_{i}}}{\int }}v^{2}\left(\hat{s}\right)d\hat{s}}$ , $X_{i}=\alpha D_{i}+\beta {\hat{v}}_{i}$ (Repnik et al. (2018)	Stroke Repnik et al. (2018)
Elevation angle	θ $=\max\left(\cos^{-1}\left(\frac{a_{vertical}}{g}\right)\right)$ Newman et al. (2017)	CP Newman et al. (2017)
Bradykinesia Index (BKI)	$BKI=\sqrt{\frac{STD*VEL}{Time_{var}*Amp_{var}}}$ , *var = STD of distances between signal peaks for time and amp* Delrobaei et al. (2016)	PD Delrobaei et al. (2016)
Movement decrement	Slope of change in amplitude Djurić-JovičićPetrovic et al. (2016); $\frac{Amplitude\;in\;2nd\;half\;time\;interval}{Amplitude\;in\;1st\;half\;time\;interval}$ Tamás et al. (2016); Fatigability index: slope of linear equation fitted with peak-to-peak angular velocities di Biase et al. (2018)	PD Tamás et al. (2016); Djurić-JovičićPetrovic et al. (2016); di Biase et al. (2018)
Velocity decrement	Compare velocities between 1st, 2nd, 3rd and 4th part of the data Garza-RodríguezSanchez-Fernandez et al. (2020); $\frac{Speed\;in\;2nd\;half\;time\;interval}{Speed\;in\;1st\;half\;time\;interval}$ Tamás et al. (2016)	PD Tamás et al. (2016); Garza-RodríguezSanchez-Fernandez et al. (2020)
Amplitude of modulation acceleration		PD Bonato et al. (2004)
Normalized mean squared error between a target signal and its forward linear prediction		PD Hoffman and McNames, (2011)

Frequency-domain	Formula/Feature description	Goal population
Dominant frequency component	Frequency associated with maximum power Hester (2006); Hssayeni et al. (2021); Frequency in 1–4Hz and 4–8 Hz bands Hoff et al. (2001)	PD Makabe and Sakamoto (2000); Hoff et al. (2001); Patel et al. (2009); Gallego et al. (2012); Budini et al. (2014); Thanawattano et al. (2015); Bravo (2017); Teufl et al. (2017); Lukšys et al. (2018); Bermeo (2019); Krishna et al. (2019); Zhu and Miller (2020); Hssayeni et al. (2021); Teufl et al. (2021)), stroke (Hester (2006))
Second dominant frequency	Frequency associated with the second highest peak Hssayeni et al. (2021)	PD Hssayeni et al. (2021)
Dominant frequency of jerk		Stroke Hester (2006), ataxia Kashyap et al. (2020)
Resonant Frequency (FR)	Peaks of FFT waveforms of angles and angular velocity Krishna et al. (2019)	Ataxia Krishna et al. (2019); Tran et al. (2020)
Energy acceleration	Energy in 0.2 Hz bin around dominant frequency Hester, (2006); Patel et al. (2010); Del Din et al. (2011); Zhang et al. (2012)	Stroke Hester, (2006); Patel et al. (2010); Del Din et al. (2011), PD (Bonato et al. (2004); Patel et al. (2009); Cole et al. (2010); Ghassemi et al. (2016), HD Bennasar et al. (2018)
Energy angular velocity		PD Rigas et al. (2016)
Energy acceleration and angular velocity	2–5 Hz: $p_{i}^{l}\left(k\right)=\overset{w\left(k\right)+sf}{\underset{n=w\left(k\right)-sf}{\sum }}\left(x_{i}^{l}\left(n\right)\right)²$ ; 5–10 Hz: $p_{i}^{h}\left(k\right)=\overset{w\left(k\right)+sf}{\underset{n=w\left(k\right)-sf}{\sum }}\left(x_{i}^{h}\left(n\right)\right)$ ; Tsipouras et al. (2012)	Stroke Zhang et al. (2012), PD Tsipouras et al. (2012), ataxia Krishna et al. (2019)
Amplitude and dominant frequency of modulation associated with acceleration		PD Bonato et al. (2004)
Average magnitude of 1st five STFT components		HD Bennasar et al. (2018)
Fractal dimension acceleration		PD Bonato et al. (2004)
Spectral power	Frequency band 1.5–3 Hz and 5–8 Hz Parnandi et al. (2010); Frequency band dyskinesia: 0.3–3 Hz Pulliam et al. (2018); Frequency band 0.5–15 Hz and 1–4 Hz Hssayeni et al. (2021); Frequency band 0.6–16 Hz Legros et al. (2004; Welch PSD of displacement; Power Spectral Density plots Bravo, (2016); Bermeo, (2019); PSD ratio 0.5–4 Hz and 4–12 Hz Ali et al. (2022); logarithm of the peak in the power spectrum in the tremor frequency band Heldman et al. (2011b)	Stroke Parnandi et al. (2010), PD Bravo, (2016); Pulliam et al. (2018); Bermeo, (2019); Hssayeni et al. (2021), dystonia Legros et al. (2004), tremor Heldman et al. (2011b); Ali et al. (2022)
Peak power	Gyroscope; 0–4 Hz frequency band Summa et al. (2017); Power of dominant frequency Hssayeni et al. (2021); Position and amplitude of dominant peaks in power spectra of acceleration signals Budini et al. (2014)	PD Jun et al. (2011); Kim et al. (2011); Summa et al. (2017); Zhu and Miller, (2020); Hssayeni et al. (2021), tremor Budini et al. (2014); Heo et al. (2015); Kwon et al. (2020); Ali et al. (2022), ataxia Gupta, (2022), MS Ketteringham, (2011)
Second peak power	Power of second dominant frequency Hssayeni et al. (2021)	PD Hssayeni et al. (2021)
Total power	Power spectrum of angular velocity Kim et al. (2011); Gyroscope; 0–4 Hz frequency band Summa et al. (2017); power ratio ACC = (Power in 3–7 Hz range)/(Power in 3–7 Hz + Power in 7–12 Hz) Schaefer et al. (2021)	PD Kim et al. (2011); Jun et al. (2011); Summa et al. (2017); di Biase et al. (2018); Schaefer et al. (2021)
Mean power	0.2–4 Hz frequency band Griffiths et al. (2012)	PD Griffiths et al. (2012)
Band power		Dyskinetic CP den Hartogvan der Krogt et al. (2022)
Spectral entropy		PD Tsipouras et al. (2012); Hssayeni et al. (2021), Ataxia Nguyen et al. (2020)
Component entropy		HD Bennasar et al. (2018)
Smoothness (Spectral Arc Length)	SAL $≜=-\overset{\Omega_{c}}{\underset{0}{\int }}\sqrt{{\left(\frac{1}{\Omega_{c}}\right)}^{2}+{\left(\frac{dV\left(\Omega \right)}{d\Omega }\right)}^{2}}\;d\omega$ , *where V*(*Ω*) $≜\frac{V\left(\Omega \right)}{V\left(0\right)}$ Newman et al. (2017)	CP Newman et al. (2017), PD Summa et al. (2017); di Biase et al. (2018)
Specific tremor index	Tremor Index (TI) = 100*(rms [TR])/(rms [A]) with TR = tremor and A = norm of angular velocity Carpinella et al. (2015); Average Tremor amplitude (ATA) = $4*LT_{s}\overset{f_{2}}{\underset{f=f_{1}}{\sum }}\sqrt{X\left(f\right)}$ with L = # of frequency bins, $T_{s}$ = sampling period, X = signal’s PSD Western et al. (2019); Mean logarithmic tremor power Benito-LeónSerrano et al. (2019); Tremor rotational amplitude and tremor amplitude based on identified peaks in power spectrum McGurrin et al. (2021)	MS Carpinella et al. (2015); Western et al. (2019), tremor (Benito-LeónSerrano et al. (2019); McGurrin et al. (2021)
Tremor Frequency (TF) and Tremor Amplitude (TA)	TF = frequency distribution for highest maxima between 1 and 15Hz; TA = square root of integral of PSD ± 1 Hz of detected frequency Šprdlík et al. (2011)	Tremor Šprdlík et al. (2011)

For the studies where both acceleration and angular velocity signals were collected, both mean and standard deviation (STD) were often calculated (Tsipouras et al., 2012; Thomas et al., 2018; Shawen et al., 2020; Hssayeni et al., 2021; Romano et al., 2021; Parnandi et al., 2010; Krishna et al., 2019; den Hartogvan der Krogt et al., 2022), as well as root-mean-square (RMS) values (van den Noort et al., 2017; Shawen et al., 2020; Habets et al., 2021; Krishna et al., 2019; den Hartogvan der Krogt et al., 2022). Additionally, mean and RMS or STD of acceleration and angular velocity separately were used in studies were one of the signals was available (Hester, 2006; Koop et al., 2006; Salarian et al., 2007; Yokoe et al., 2009; Patel et al., 2010; Zwartjes et al., 2010; Del Din et al., 2011; Griffiths et al., 2012; Strohrmann et al., 2013; Budini et al., 2014; Lee et al., 2015b; Heo et al., 2015; Djurić-JovičićPetrovic et al., 2016; Ghassemi et al., 2016; Samotus et al., 2016; Angeles et al., 2017; Bennasar et al., 2018; López-Blanco et al., 2018; Cavallo et al., 2019; Garza-RodríguezSanchez-Fernandez et al., 2020; Kwon et al., 2020; Li et al., 2020; Dominguez-Vega et al., 2021; Habets et al., 2021; Oubre et al., 2021; Schaefer et al., 2021; Gupta, 2022), as well as median angular velocity in one study (Garza-RodríguezSanchez-Fernandez et al., 2020). Maximal linear velocity was additionally often used as key feature, mostly by integration of the acceleration signal (Hester, 2006; Okuno et al., 2006; Yokoe et al., 2009). The range of angular displacement or range of motion was only used in studies in PD (Espay et al., 2011; Jun et al., 2011; Kim et al., 2011; Tamás et al., 2016; Kwon et al., 2018; Cavallo et al., 2019), mainly to assess hypokinesia. The range of acceleration and angular velocity was included in PD (Patel et al., 2009; Yokoe et al., 2009; Rabelo et al., 2017; di Biase et al., 2018; Shawen et al., 2020; Habets et al., 2021; Otten et al., 2015), tremor (Oubre et al., 2021) and CP (Strohrmann et al., 2013; Newman et al., 2017), as well as the inter-quartile range for PD (Garza-RodríguezSanchez-Fernandez et al., 2020; Habets et al., 2021). The range of jerk and angular acceleration was used in one study in PD (Spasojević et al., 2017).

Peak-to-peak and magnitude of angular velocity were additionally used in PD (Summa et al., 2017; Pulliam et al., 2018; di Biase et al., 2018; Hssayeni et al., 2021), whereas Repnik and others calculated a rotational jerk index for angular velocity values to evaluate hand rotation in stroke (Repnik et al., 2018). Finally, a study in PD used the square root of the sum of squares of jerk signals and named this feature ‘segment velocity’ (Keijsers et al., 2003).

As basis statistical features, kurtosis and skewness were popular in PD (Parnandi et al., 2010; Ghassemi et al., 2016; Lonini et al., 2018; Thomas et al., 2018; Shawen et al., 2020; Hssayeni et al., 2021), but not in other populations apart from one ataxia study (Gupta, 2022). With respect to signal dynamics, multiple forms of entropy were used, most commonly sample entropy and approximate entropy in PD (Patel et al., 2009; Chelaru et al., 2010; Tsipouras et al., 2012; Ghassemi et al., 2016; Liu et al., 2016; Lonini et al., 2018; Shawen et al., 2020), stroke (Patel et al., 2010) and ataxia (Kashyap et al., 2020) and Shannon entropy and permutation entropy in PD (Hssayeni et al., 2021), dyskinetic CP (den Hartogvan der Krogt et al., 2022) and HD (Bennasar et al., 2018). Fuzzy entropy was additionally calculated in two ataxia studies (Nguyen et al., 2020; Tran et al., 2020). Apart from entropy, the Gini index and Lyapunov exponent were additionally used as a measure of signal complexity in PD (Hssayeni et al., 2021), ataxia (Nguyen et al., 2020) and HD (Bennasar et al., 2018). The same HD study additionally used recurrence rate, determinism and average diagonal line to evaluate signal dynamics (Bennasar et al., 2018).

For signal smoothness, RMS of jerk was often used as a straightforward measure in PD (Parnandi et al., 2010) and stroke (Knorr, 2005; Hester, 2006), as well as a jerk metric for which multiple definitions were given, mostly RMS jerk normalized over time/peak velocity or mean jerk (Newman et al., 2017; Zhang et al., 2012; Lonini et al., 2018; Habets et al., 2021; Romano et al., 2021; Hester, 2006; Carpinella et al., 2014; den Hartogvan der Krogt et al., 2022; Bai et al., 2021). Additionally, smoothness measures were also described as the difference between movement accelerometer readings and smoothed readings, number of movement units or number of speed peaks (Kamper et al., 2002; Otten et al., 2015; Bai et al., 2021).

Coefficient of variation was often used as a measure of variability or rhythm for different signals such as excursion angle (Heldman et al., 2011a; Espay et al., 2011; Tamás et al., 2016; Kwon et al., 2018), (angular) velocity (Espay et al., 2011; Lee et al., 2015b; Kwon et al., 2018), amplitude (Lee et al., 2015b; Djurić-JovičićPetrovic et al., 2016) and movement frequency (Lee et al., 2015b; Cavallo et al., 2019; Tran et al., 2020), while two studies in PD defined ‘rhythm’ *via* the STD of intervals of a finger tapping movement (Okuno et al., 2006) and any sequence of regularly occurring events (Martinez-Manzanera et al., 2016). Finally, a stroke study defined variability as the RMS error between a reference trial and a warped trial (Thies et al., 2009). Considering the geometrical structure of a non-linear time-series, Newman et al. included Higuchu’s fractional dimension in children with CP (Newman et al., 2017).

With respect to orientation and rotational information, correlation between the different axes of the accelerometer or gyroscope was often included as a feature in PD (Lonini et al., 2018; Shawen et al., 2020; Zhu and Miller, 2020; Habets et al., 2021), HD (Bennasar et al., 2018) and stroke (Hester, 2006). Additionally, the peak of the normalized cross-correlation from pairs of acceleration time series and the lag of first peak in autocorrelation acceleration were included in two PD studies (Patel et al., 2009; Cole et al., 2010). Concerning trajectories and travelled distances, multiple studies used different definitions for this feature. 3D hand trajectory and length of 3D trajectory (van Meulen et al., 2015) and path-length ratio were used in stroke (Kamper et al., 2002), while the index of curvature (deviation from a straight line) was used in dyskinetic CP (Sanger, 2006). Elevation angle was included in a CP study, while in stroke, the similarity of hand trajectories was used (Newman et al., 2017; Repnik et al., 2018). Two studies in patients with ataxia used mean and standard deviation of Euclidian distance from the mean trajectory and curved and straight-line similarity analysis (Martinez-Manzanera et al., 2018; Dominguez-Vega et al., 2021). In PD, Heldman et al. used a bradykinesia index, based on variability in time and amplitude of task execution whereas Tamas et al. and Garza-Rodriguez and others quantified hypokinesia using velocity decrement, which is defined as a decrease in velocity between subsequent data parts (Tamás et al., 2016; Garza-RodríguezSanchez-Fernandez et al., 2020).

In the frequency-domain, the dominant frequency component of acceleration/angular velocity or both was most often used (Makabe and Sakamoto, 2000; Hoff et al., 2001; Hester, 2006; Patel et al., 2009; Gallego et al., 2012; Budini et al., 2014; Thanawattano et al., 2015; Bravo, 2017; Teufl et al., 2017; Lukšys et al., 2018; Bermeo, 2019; Krishna et al., 2019; Zhu and Miller, 2020; Hssayeni et al., 2021; Teufl et al., 2021), while only three studies included the second dominant frequency or dominant frequency of jerk (Hester, 2006; Kashyap et al., 2020; Hssayeni et al., 2021). Energy in the frequency spectrum was often included in multiple populations, both for the acceleration signal (Bonato et al., 2004; Hester, 2006; Patel et al., 2009; Cole et al., 2010; Patel et al., 2010; Del Din et al., 2011; Ghassemi et al., 2016), angular velocity signal (Rigas et al., 2016) or both (Tsipouras et al., 2012; Zhang et al., 2012; Krishna et al., 2019). One PD study additionally included amplitude and dominant frequency of modulations associated with the acceleration signal as well as fractal dimension (Bonato et al., 2004), while one HD study included the average magnitude of the first five Short-Term-Fourier-Transfer components (Bennasar et al., 2018). Apart from the frequency, power in specific frequency bands was a popular feature in multiple populations, including spectral power (Legros et al., 2004; Parnandi et al., 2010; Heldman et al., 2011b; Bravo, 2016; Pulliam et al., 2018; Bermeo, 2019; Hssayeni et al., 2021; Ali et al., 2022), peak power (Makabe and Sakamoto, 2000; Jun et al., 2011; Kim et al., 2011; Summa et al., 2017; Hssayeni et al., 2021), total power (Kim et al., 2011; Jun et al., 2011; Summa et al., 2017; di Biase et al., 2018; Zhu and Miller, 2020; Ali et al., 2022; Budini et al., 2014; Heo et al., 2015; Kwon et al., 2020; Ketteringham, 2011; Gupta, 2022), mean power (Griffiths et al., 2012) and band power (den Hartogvan der Krogt et al., 2022). Considering entropy in the frequency domain, spectral entropy was used in two PD studies (Tsipouras et al., 2012; Hssayeni et al., 2021) and one ataxia study (Nguyen et al., 2020), as well as component entropy in HD (Bennasar et al., 2018). Spectral Arc Length was used as a measure of smoothness in two PD studies and one CP study (Newman et al., 2017; Summa et al., 2017; di Biase et al., 2018). For tremor studies, tremor frequency and tremor amplitude (Šprdlík et al., 2011) were included as well as multiple specific tremor indices: Carpinella et al. defined the tremor index as the ratio of tremor (defined by peaks in the frequency spectrum) and the norm of angular velocity (Carpinella et al., 2015). Western et al. defined average tremor amplitude as the product of frequency bins, sampling period and the signal’s power spectral density (Western et al., 2019), whereas Benito-Leon et al. and McGurrin et al. used the mean logarithmic tremor power and tremor rotational amplitude/amplitude respectively, based on identified peaks in the power spectrum (Benito-LeónSerrano et al., 2019; McGurrin et al., 2021).

### Statistical method used

**FIGURE 4:**
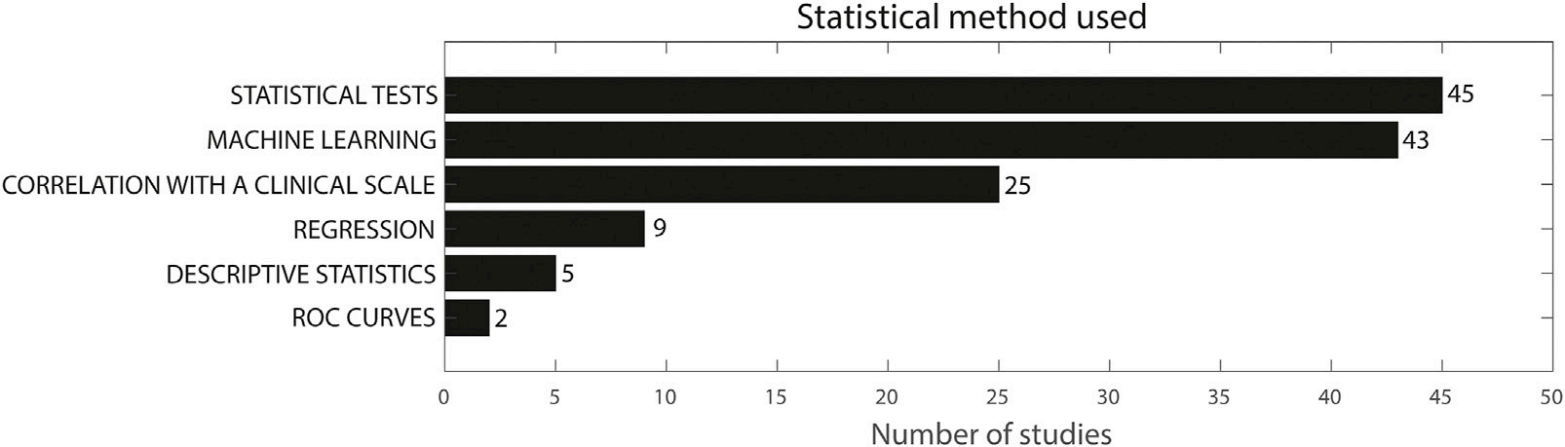
Statistical method used in the included studies. The sum does not add up to 101 because multiple studies used more than one methodology.

Figure 4 gives a representation of the statistical methods used in the included studies. Forty-five studies included between- or within-group comparisons using statistical tests, (Makabe and Sakamoto, 2000; Hoff et al., 2001; Kamper et al., 2002; Legros et al., 2004; Okuno et al., 2006; Sanger, 2006; Thies et al., 2009; Chelaru et al., 2010; Espay et al., 2011; Kim et al., 2011; Jun et al., 2011; Šprdlík et al., 2011; Griffiths et al., 2012; Tsipouras et al., 2012; Strohrmann et al., 2013; Budini et al., 2014; Carpinella et al., 2014; Carpinella et al., 2015; Heo et al., 2015; Lee et al., 2015; Rahimi et al., 2015; Thanawattano et al., 2015; Delrobaei et al., 2016; Liu et al., 2016; Samotus et al., 2016; Summa et al., 2016; Tamás et al., 2016; Newman et al., 2017; Rabelo et al., 2017; Delrobaei et al., 2018; di Biase et al., 2018; Kwon et al., 2018; Lukšys et al., 2018; Repnik et al., 2018; Li et al., 2020; Nguyen et al., 2020; Bai et al., 2021; Habets et al., 2021; Romano et al., 2021; Schaefer et al., 2021; Ali et al., 2022; Chan et al., 2022; Gupta et al., 2022), mainly parametric and non-parametric ANOVA and parametric and non-parametric t-tests, whereas 43 studies used machine learning (Keijsers et al., 2003; Bonato et al., 2004; Hester et al., 2006; Patel et al., 2009; Chelaru et al., 2010; Cole et al., 2010; Parnandi et al., 2010; Patel et al., 2010; Zwartjes et al., 2010; Del Din et al., 2011; Heldman et al., 2011; Gallego et al., 2012; Griffiths et al., 2012; Tsipouras et al., 2012; Zhang et al., 2012; Otten et al., 2015; Ghassemi et al., 2016; Martinez-Manzanera et al., 2016; Rigas et al., 2016; Spasojević et al., 2017; Angeles et al., 2017; Rabelo et al., 2017; Lonini et al., 2018; Martinez-Manzanera et al., 2018; Pulliam et al., 2018; Thomas et al., 2018; Garza-Rodríguez et al., 2018; Bermeo et al., 2019; Cavallo et al., 2019; Krishna et al., 2019; Kashyap et al., 2020; Shawen et al., 2020; Tran et al., 2020; Hssayeni et al., 2021; Park et al., 2021; Habets et al., 2021; Teufl et al., 2021; Dominguez-Vega et al., 2021; Oubre et al., 2021; Ali et al., 2022; den Hartog et al., 2022). Twenty-five studies evaluated correlation of sensor features with clinical scales (Newman et al., 2017; Repnik et al., 2018; Okuno et al., 2006; Salarian et al., 2007; Yokoe et al., 2009; Kim et al., 2011; Jun et al., 2011; Delrobaei et al., 2016; Liu et al., 2016; Djurić-JovičićPetrovic et al., 2016; di Biase et al., 2018; Delrobaei et al., 2018; Benito-LeónSerrano et al., 2019; McGurrin et al., 2021; Chan et al., 2018; López-Blanco et al., 2018; Kwon et al., 2020; Teufl et al., 2017; Western et al., 2019; Krishna et al., 2019; Tran et al., 2020; Nguyen et al., 2020; Oubre et al., 2021; Gupta, 2022; Kim et al., 2018), whereas nine studies used regression analysis for a similar purpose (Knorr, 2005; Sanger, 2006; Yokoe et al., 2009; Heldman et al., 2011a; Šprdlík et al., 2011; Strohrmann et al., 2013; van Meulen et al., 2015; Oubre et al., 2021; Ali et al., 2022). Finally, five studies used only descriptive statistics or observation without hypothesis testing (van den Noort et al., 2017; Bravo, 2016; Bravo, 2017; Zhu and Miller, 2020; Ketteringham, 2011) and two studies evaluated ROC curves (Hoffman and McNames, 2011; Oubre et al., 2021). The sum of these numbers does not add up to 101, because multiple studies used multiple of the abovementioned methods. Eleven studies used both statistical tests for comparison between groups and correlation with a clinical scale (Newman et al., 2017; Repnik et al., 2018; Kim et al., 2011; Jun et al., 2011; Delrobaei et al., 2016; Liu et al., 2016; Djurić-JovičićPetrovic et al., 2016; di Biase et al., 2018; Delrobaei et al., 2018; Nguyen et al., 2020; Gupta, 2022), whereas five studies used statistical tests and machine learning (Chelaru et al., 2010; Griffiths et al., 2012; Tsipouras et al., 2012; Rabelo et al., 2017; Habets et al., 2021). Yokoe et al. used both logistic regression and correlation with a clinical scale in PD (Yokoe et al., 2009). In patients with ataxia, Tran et al. used statistical tests, correlation with a clinical scale and machine learning (Tran et al., 2020) and Oubre et al. used statistical tests, regression, correlation with a clinical scale and machine learning (Oubre et al., 2021). In participants with essential tremor, Ali et al. used statistical tests, regression analysis and machine learning (Ali et al., 2022) and Sprdlik et al. used statistical tests, ROC curves and regression analysis (Šprdlík et al., 2011). In CP, Sanger et al. used both regression analysis and ANOVA/t-tests (Sanger, 2006), and Strohrmann et al. used a *t*-test, to subsequently continue with a linear regression for the features that were significantly different between groups (Strohrmann et al., 2013).

### Sensitivity and/or responsiveness of most prevalent sensor features

Table 3 provides an overview of the features included by more than five articles, the number of articles reporting sensitivity of the specific feature and the number of articles that identified a significant difference between groups, severity levels or pre/post intervention.

**TABLE 3:** Most prevalent features, the number of articles they appear in, the number of articles reporting sensitivity and the number of articles reporting significant results.

Feature	Articles (n)	Articles reporting sensitivity (n)	Articles reporting significant results (n)
RMS angular velocity	18	12	11
Mean acceleration	17	3	2
Execution time	17	10	10
Dominant frequency domain	15	1	1
Peak power	12	6	6
Sample entropy	11	2	2
RMS angular displacement/movement amplitude	11	10	9
Energy acceleration in frequency domain	11	0	0
STD acceleration	11	0	0
RMS acceleration	10	1	1
Mean angular velocity	10	6	6
Jerk metric	9	5	4
Coefficient of variation	8	5	5
Spectral power	7	4	3
Range acceleration	7	2	1
Approximate entropy	7	2	2
Range angular displacement	6	4	4
Mean acceleration and angular velocity	6	1	1
Correlation between axes	6	1	0
Skewness	6	0	0
Kurtosis	6	0	0

RMS of angular velocity was reported in 18 studies, with sensitivity results for 11 studies. In PD, Van den Noort et al. found significantly higher RMS values for ON vs. OFF dopaminergic medication, while Summa et al. did not find a significant difference between medication states (van den Noort et al., 2017; Summa et al., 2017). Espay et al. found 25% improvement in RMS values after dopaminergic medication in PD patients (Espay et al., 2011). Kwon et al. and Luksys et al. found significant higher RMS angular velocity values for PD patients in comparison with controls, whereas two studies found a correlation of -0.78 between RMS angular velocity and clinical scores of the UPDRS (Jun et al., 2011; Kim et al., 2011; Kwon et al., 2018; Lukšys et al., 2018). Additionally, Heldman et al. found a correlation of -0.78 between RMS angular velocity values and the modified bradykinesia rating scale (Heldman et al., 2011a) and Salarian et al. found good correlation between RMS angular velocity values and the UPDRS bradykinesia subscore, as well as good correlation between RMS angular velocity of the roll axis and the tremor subscore of the UPDRS (Salarian et al., 2007). In patients with tremor, spearman correlation between RMS angular velocity and tremor severity scores ranged from 0.19 (finger-to-nose) to 0.73 (keeping arms extended in front of the body) for Lopez-Bianco et al. (López-Blanco et al., 2018) and between 0.41 and 0.70 for Kwon et al. (Kwon et al., 2020), whereas Heo et al. found lower RMS angular velocity values after electrical stimulation (Heo et al., 2015).

Seventeen studies reported mean acceleration as a feature, but only two PD studies and one ataxia study discussed its sensitivity. Romano et al. found lower mean acceleration for PD patients in comparison with the control group, while Zwartjes et al. did not find significant differences between ON and OFF stimulation states of deep brain stimulation (Zwartjes et al., 2010; Romano et al., 2021). In patients with Ataxia, Samotus et al. found lower mean acceleration after botulinum-toxin-A injections (Samotus et al., 2016). Execution time was included in 17 studies, with reported sensitivity for 11 studies. Execution time significantly differed between different severity levels (Repnik et al., 2018) and between healthy controls and patients with stroke (Thies et al., 2009; Repnik et al., 2018) and MS (Carpinella et al., 2014; Carpinella et al., 2015) and between the paretic and non-paretic arm in children with unilateral CP (Newman et al., 2017). Execution time was significantly longer for PD patients in comparison with healthy controls (Rabelo et al., 2017; di Biase et al., 2018) and for patients with multiple system atrophy of parkinsonian type and progressive supranuclear palsy in comparison with healthy controls (Djurić-JovičićPetrovic et al., 2016). Third, execution time was significantly different between the ON and OFF medication state in PD patients (van den Noort et al., 2017). In CP, execution time was one of the three features to best estimate upper limb performance in a regression analysis (Strohrmann et al., 2013).

The dominant frequency domain was included in 15 studies, but only Hoff et al. reported individual contributions of this feature, reporting that amplitude in 1–4Hz and 4–8 Hz frequency bands correlated with the modified Abnormal Involuntary Movement Scale (Hoff et al., 2001). Peak power was included in 12 studies, of which six discussed its individual sensitivity. Jun et al. reported a good correlation between peak power and clinical bradykinesia scores and Kim et al. reported decreasing peak powers with increasing UPDRS scales steps (Jun et al., 2011; Kim et al., 2011), while Makabe et al. reported increasing peak powers with increasing severity stages of the Hoehn and Yahr scale (Makabe and Sakamoto, 2000). Similarly, Summa et al. reported increases in peak power in ON vs. OFF medication state (Summa et al., 2017). In essential tremor, Heo et al. reported higher peak power after electrical stimulation (Heo et al., 2015) and Kwon et al. reported high correlation between peak power and tremor severity scores (Kwon et al., 2020).

Sample entropy was included in 11 studies, but only two PD studies reported its sensitivity. Chelaru et al. found significantly higher entropy for dyskinetic PD patients in comparison with non-dyskinetic PD patients, as well as Liu et al. who found a significant difference between PD patients and healthy controls and good correlation with UPDRS scores (Chelaru et al., 2010; Liu et al., 2016). RMS of angular displacement was included in 11 studies, of which ten reported sensitivity. Tamas et al. found significant differences in RMS amplitude before and after subthalamic stimulation and Espay et al. found significant differences between ON and OFF medication state in PD (Espay et al., 2011; Tamás et al., 2016). Kwon et al. found significantly lower RMS amplitudes for PD patients in comparison with controls and Jun et al. found decreasing angular displacement with increasing bradykinesia scores, but this was based on visual observation (Jun et al., 2011; Kwon et al., 2018). Chan et al. found higher values for angular displacement for patients with PD with tremor in comparison with essential tremor (Chan et al., 2022). Kim et al. additionally found a significant difference between PD patients and controls (Kim et al., 2011), whereas Heldman et al. found a correlation of −0.81 between RMS excursion angle and clinical scores (Heldman et al., 2011a). Delrobaei et al. found a higher tremor severity score (which was composed of the RMS values of angular velocity) for tremor-dominant PD patients in comparison with non-tremor dominant PD patients and good correlation between tremor severity score and UPDRS scores (Delrobaei et al., 2018). In essential tremor, Kwon et al. and Chan et al. found correlations ranging from 0.29–0.66 and 0.80–0.93 respectively, between RMS angular displacement and tremor severity scores (Chan et al., 2018; Kwon et al., 2020). Energy and STD of acceleration were included in 10 studies, but none reported sensitivity.

RMS of acceleration was included in 10 studies, but only van den Noort et al. discussed its specific contribution in PD patients, reporting increased RMS acceleration in ON vs. OFF medication state during a finger tapping and opening/closing of the hand task (van den Noort et al., 2017). Mean angular velocity was also included in 10 studies with six of them reporting sensitivity. In PD, three studies found lower mean angular velocity for PD patients in comparison with healthy controls (Lee et al., 2015b; Djurić-JovičićPetrovic et al., 2016; Romano et al., 2021), whereas one study additionally identified significant differences between ON/OFF DBS stimulation (Salarian et al., 2007). Garza-Rodriguez et al. found lower angular velocity values for PD patients with higher clinical severity (Garza-RodríguezSanchez-Fernandez et al., 2018). In patients with ataxia, Oubre et al. found significant differences between patients and healthy controls (Oubre et al., 2021).

Jerk metrics were calculated in nine studies with five reporting on its sensitivity. Romano et al. used the dimensionless jerk index as a jerk metric and found a significant difference between PD patients and healthy controls, while Habets et al. did not find a significant difference between ON and OFF medication state in PD patients (Habets et al., 2021; Romano et al., 2021). Carpinella et al. found a significantly higher jerk measure for patients with MS in comparison with healthy controls and a negative correlation between the jerk measure and ARAT score (r = −0.90) (Carpinella et al., 2014). In children with unilateral CP, Newman and others found a significantly higher normalised jerk index for the paretic arm in comparison with the non-paretic arm, but no correlation with the Melbourne Assessment Scale (Newman et al., 2017). In children with spasticity, the normalized jerk score improved significantly after botulinum-toxin A injections (Bai et al., 2021).

Coefficient of variation (CoV) was included in eight studies, where CoV of time and amplitude was mostly calculated to evaluate bradykinesia. Djuric-Jovicic and others found significant differences between PD patients and healthy controls for both CoV of time and amplitude, whereas Lee et al. found significant differences for CoV of speed, amplitude and frequency between PD patients and controls (Lee et al., 2015b; Djurić-JovičićPetrovic et al., 2016). Kwon et al. additionally found significant differences between PD patients and controls for the CoV of angles and velocity (Kwon et al., 2018). Tamas et al. found that the coefficient of variation—also called ‘rhythm’—improved significantly after bilateral and contralateral subthalamic stimulation, whereas Espay et al. found significant differences between ON and OFF medication state for CoV in PD patients (Espay et al., 2011; Tamás et al., 2016).

Spectral power was used in seven studies of which four reported sensitivity. Bravo et al. compared power spectral density (PSD) plots between PD patients and healthy controls and found both higher and lower PSD amplitude for PD patients in comparison with healthy controls, depending on the individual (Bravo, 2016). In patients with dystonia, Legros et al. found a decrease of the area under the spectrum curve after deep brain stimulation surgery (Legros et al., 2004). Ali et al. found higher PSD ratios for patients with essential tremor in comparison with healthy controls (Ali et al., 2022), whereas Heldman et al. found correlations from 0.77–0.83 between the logarithm of peak power and the UPDRS scores (Heldman et al., 2011b). The range of acceleration was additionally calculated in seven articles, but only two articles reported its sensitivity. Rabelo et al. found a significantly higher acceleration range for healthy controls in comparison with PD patients, while Habets et al. did not find a significant difference between ON and OFF medication state in PD patients (Rabelo et al., 2017; Habets et al., 2021). Approximate entropy was also included in seven studies, but only two PD studies included its sensitivity, where Liu et al. and Luksys et al. found significant differences between PD patients and a control group (Liu et al., 2016; Lukšys et al., 2018).

Range of angular displacement was calculated in six studies, but only four discussed its sensitivity. Djuric et al. reported a higher range for healthy controls in comparison with PD patients, whereas van den Noort et al. reported lower displacement in the ON vs. OFF medication state and improved amplitude in the ON compared to OFF state (van den Noort et al., 2017; Djurić-JovičićPetrovic et al., 2016). Romano et al. found significant differences between PD patients and healthy controls for wrist flexion and shoulder movements and Salarian et al. found significantly lower angular displacements at the level of the wrist for PD patients compared to healthy controls (Salarian et al., 2007; Romano et al., 2021). Energy of acceleration in the frequency domain and STD of acceleration were included in 11 articles, but all of them included these features as part of a feature set for machine learning, without discussing its individual contribution.

Mean acceleration and angular velocity were included in six studies, but only Romano et al. found significantly lower mean acceleration and angular velocity in PD patients in comparison with healthy controls (Romano et al., 2021). Correlation between axes was included in six studies, but only Zhu et al. reported no significant differences in correlations when comparing PD patients in ON and OFF medication state (Zhu and Miller, 2020). Kurtosis and skewness were additionally included in six studies, but none of them reported the contribution of the individual features.

### Clinical application

**FIGURE 5:**
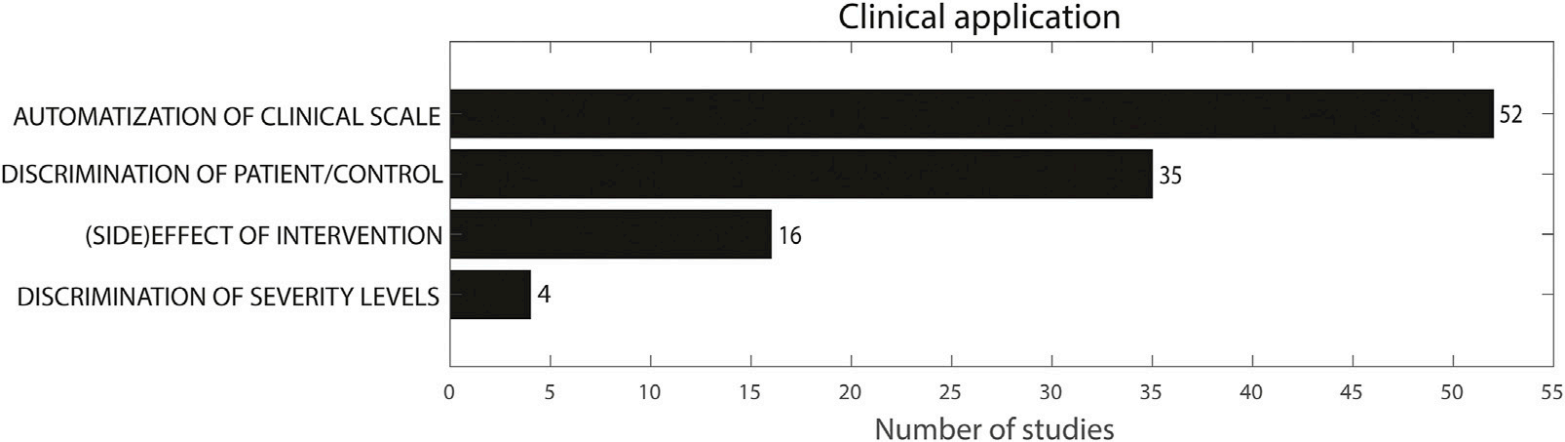
Clinical application. The sum does not add up to 101 because multiple studies used more than one methodology.

Figure 5 presents an overview of the clinical application of the included studies. Fifty-two studies used sensor features for the automatization of a clinical scale (Bonato et al., 2004; Knorr, 2005; Hester, 2006; Okuno et al., 2006; Salarian et al., 2007; Patel et al., 2009; Yokoe et al., 2009; Cole et al., 2010; Parnandi et al., 2010; Patel et al., 2010; Zwartjes et al., 2010; Del Din et al., 2011; Heldman et al., 2011a; Jun et al., 2011; Kim et al., 2011; Gallego et al., 2012; Griffiths et al., 2012; Zhang et al., 2012; Strohrmann et al., 2013; Carpinella et al., 2014; van Meulen et al., 2015; Otten et al., 2015; Delrobaei et al., 2016; Martinez-Manzanera et al., 2016; Rigas et al., 2016; Spasojević et al., 2017; Teufl et al., 2017; Bennasar et al., 2018; Chan et al., 2018; Delrobaei et al., 2018; Garza-Rodríguez et al., 2018; Kim et al., 2018; Lonini et al., 2018; López-Blanco et al., 2018; Pulliam et al., 2018; Thomas et al., 2018; Benito-Leónet al., 2019; Bermeo, 2019; Cavallo et al., 2019; Krishna et al., 2019; Western et al., 2019; Garza-Rodríguez et al., 2020; Kwon et al, 2020; Nguyen et al., 2020; Shawen et al., 2020; Hssayeni et al., 2021; McGurrin et al., 2021; Oubre et al., 2021; Park et al., 2021; Teufl et al., 2021; den Hartog et al., 2022). Sixteen studies used sensor features to evaluate the effect of an intervention (Hoff et al., 2001; Keijsers et al., 2003; Legros et al., 2004; Koop et al., 2006; Chelaru et al., 2010; Espay et al., 2011; Budini et al., 2014; Heo et al., 2015; Rahimi et al., 2015; Bravo, 2016; Samotus et al., 2016; Tamás et al., 2016; Angeles et al., 2017; Zhu and Miller, 2020; Bai et al., 2021; Habets et al., 2021), whereas 35 studies used the obtained features to discriminate between patients and controls or between different patient groups (van den Noort et al., 2017; Newman et al., 2017; Sanger, 2006; Repnik et al., 2018; Hoffman and McNames, 2011; Tsipouras et al., 2012; Lee et al., 2015b; Delrobaei et al., 2016; Ghassemi et al., 2016; Liu et al., 2016; Djurić-JovičićPetrovic et al., 2016; Spasojević et al., 2017; Summa et al., 2017; Rabelo et al., 2017; Kwon et al., 2018; di Biase et al., 2018; Li et al., 2020; Romano et al., 2021; Bravo, 2017; Chan et al., 2022; Delrobaei et al., 2018; Makabe and Sakamoto, 2000; Schaefer et al., 2021; Thanawattano et al., 2015; Lukšys et al., 2018; Ali et al., 2022; Šprdlík et al., 2011; Thies et al., 2009; Carpinella et al., 2015; Ketteringham, 2011; Tran et al., 2020; Dominguez-Vega et al., 2021; Gupta, 2022; Martinez-Manzanera et al., 2018; Kashyap et al., 2020). Four studies subsequently discriminated between different severity levels (Kamper et al., 2002; Delrobaei et al., 2016; Spasojević et al., 2017; Repnik et al., 2018). Again, there was some overlap in clinical applications: Delrobaei et al., Spasojevic et al. and Repnik et al. compared control and patient groups as well as severity levels within the patient group, while also correlating sensor features with a clinical scale (Delrobaei et al., 2016; Spasojević et al., 2017; Repnik et al., 2018). Kamper et al. compared a patient and control group but also compared severity levels separately (Kamper et al., 2002).

## Discussion

The primary objective of this scoping review was to provide an overview of sensor set-up and type, included tasks, sensor features and statistical methods that are used to evaluate movement disorders during upper limb tasks in multiple pathological populations. We identified 101 studies in eight pathological conditions using wearable sensors placed on the upper limb during upper limb tasks and including at least one sensor feature based on linear acceleration or angular velocity. Of all included studies, 55% were studies in PD, 12% were studies with essential tremor patients, 11% were studies in stroke patients, 8% were studies in adults or children with ataxia, 6% were studies including participants with MS and 5% included children with CP. Adults with HD and spasticity and dystonia in children represented only 1% of the included studies. When comparing these numbers with the prevalence of the abovementioned conditions, an important imbalance emerges. Worldwide, approximately 101 million people are living post-stroke (Feigin et al., 2022), 25 million people live with essential tremor (Song et al., 2021), 17 million people live with CP (McIntyre et al., 2011), 10 million people are estimated to live with PD (Van Den Eeden, 2003; Okubadejo et al., 2006; Marras et al., 2018), approximately 0.2–3 million people live with ataxia, depending on the type (Musselman et al., 2014; Ruano et al., 2014) and 0.2 to 0.5 million people live with HD, depending on the geographical area (Crowell et al., 2021; Medina et al., 2022). While stroke is much more prevalent than PD or essential tremor, this ratio is not reflected in the number of available studies per condition. More surprisingly, where CP is the most prevalent neurological childhood condition included, its high prevalence does not correspond with the number of studies investigating the associated movement disorders using wearable sensors. Current findings thus identify a major gap between prevalence of a condition and insights in the related movement disorders. Especially for early-onset conditions such as CP, more insights in the disturbed movement patterns from an early age could benefit targeted therapy and long-term treatment management.

The abundance of included PD studies reflects its more advanced state-of-the-art assessment in comparison with other pathological populations. These insights offer opportunities and learning experiences for clinicians and researchers aiming to bridge the gap between technology and clinical measures in the quantitative evaluation of movement disorders. Although wide-spread in research, the clinical implementation of IMU-based analysis of movement disorders is lacking in clinical practice in all populations, mainly due to the lack of validation of algorithms in real-world conditions (Del Din et al., 2021).

With respect to sensor type, IMUs containing both an accelerometer and gyroscope were most often used, where a time-related trend was clearly visible in the included PD studies: between 2000 and 2010, all PD studies included either an accelerometer or a gyroscope, whereas after 2010, IMUs were almost exclusively used. This trend is presumably supported by technological advancements, allowing more sensors in a smaller device with longer battery life combined with more affordable prices for IMUs.

Sensor location, number of included sensors and upper limb tasks were separately discussed to provide a comprehensive overview. However, conclusions should be drawn on a combination of these settings as they are closely inter-related. E.g., all but one of the nine studies that placed one sensor on the index finger included the finger tapping (Okuno et al., 2006; Heldman et al., 2011b; Hoffman and McNames, 2011; Kim et al., 2011; Tamás et al., 2016) or finger-to-nose task (Thanawattano et al., 2015; Bravo, 2017; Zhu and Miller, 2020) and of the nine studies who placed a sensor one the thumb and index finger, all included finger tapping (Yokoe et al., 2009; Heldman et al., 2011a; Espay et al., 2011; Lee et al., 2015b; Djurić-JovičićPetrovic et al., 2016; Liu et al., 2016; Summa et al., 2017; Li et al., 2020; Park et al., 2021). Finger tapping, finger-to-nose, wrist pro/supination and opening/closing hand were the only tasks included in studies with sensors solely on the index finger and/or thumb and all of those were in PD patients. From all included studies, 68 placed a sensor on the wrist and/or forearm and 35 on the dorsal hand but out these 35, 22 placed a sensor on both the hand and wrist. The more proximal sensor placement of hand, wrist and forearm was used in all pathologies and in combination with more functional tasks such as drinking, writing and eating. Additionally, the four studies that measured activities in a home environment all placed sensors on the wrist, mostly likely due to the high comfort and ease of use of wrist-worn sensors (Griffiths et al., 2012; Habets et al., 2021; Gupta, 2022; den Hartogvan der Krogt et al., 2022). When selecting a specific sensor set-up, one should thus carefully consider whether the aim is to only automate a clinical scale, or to evaluate movement disorders during a range of functional tasks. For the latter, one sensor on the hand, wrist or forearm could be sufficient to maximise adherence and wide applicability.

The collection of upper limb tasks included in the selected studies reflects the insight that the choice of upper limb task is heavily dependent on the movement disorder. The high prevalence of finger tapping and wrist pro/supination in the PD studies follows from their presence in the Motor Examination part of the (MDS-)UPDRS (Goetz et al., 2008), whereas the finger-to-nose task and keeping arms extended in front of the body are part of both the (MDS-)UPDRS and the Essential Tremor Rating Assessment Scale (Elble et al., 2012). Both tasks are well-suited to quantify decrease and slowness of movements, corresponding with the clinical symptoms of hypokinesia and bradykinesia in PD. Since the (MDS)-UPDRS and Essential Tremor Assessment Scale are well implemented in clinical practice, patients are often requested to perform these tasks in the presence of a neurologist, facilitating combination of this clinical appointment with research purposes. In stroke, the Wolf Motor Function Task was most popular, presumably because this scale is used in daily practice for the evaluation of upper extremity rehabilitation progress. An important notion is that the aetiological differences between PD/tremor on the one hand and CP, stroke and dystonia on the other hand influence the potential of task execution. In CP and stroke, functional ability can be impaired to a level where execution of specific functional tasks is not possible, which requires a very different approach in comparison with PD or tremor, where most tasks can be executed but performance may be impaired. When the level of physical impairment prohibits the execution of specific tasks, one should focus on monitoring of the movement disorders during home-based activities such as powered mobility (e.g. joy-stick steering) or in rest positions in the case of severe CP or stroke (den Hartogvan der Krogt et al., 2022).

In the case of severe functional impairment occurring in e.g. dystonia or spasticity, there are some extra challenges with respect to sensor adherence and reliability of data streams which need to be taken into account. Sensor fixation should be sufficiently tight in the case of severe movement disorders, to avoid sensor dislodgement and subsequent data loss. From the studies involved in current selection, only one study included participants with such severe movement disorders that they were only evaluated during rest or power mobility driving since other tasks were impossible (den Hartogvan der Krogt et al., 2022). This specific study did not report any information on data loss apart from the fact that linear interpolation from adjacent time stamps was used in case of missing data stamps. There are multiple reviews discussing the use of wearable sensors for the detection, of motor symptoms in e.g. HD and PD, but none of those mention missing data or data loss of the included studies (Maetzler et al., 2013; Tortelli et al., 2021). To allow quality control, future studies measuring in natural environments and for longer duration should discuss missing data and data loss more thoroughly.

The secondary objective was to identify the most sensitive sensor features for symptom detection and quantification and describe the application of the proposed methods in clinical practice. Similar to the requested tasks, the derived sensor features were dependent of the movement disorder under investigation. Mean amplitude, movement/amplitude decrement and RMS, range and IQR of angular displacement were only used in PD studies and are hypothesized to correlate with the definition of hypokinesia (reduction in movement amplitude) in the (MDS-)UPDRS. Range and RMS of angular displacement can detect differences between PD and TD groups and quantify the severity of hypokinesia, implying that these features can be used in clinical practice as simply interpretable triggers of movement reduction. Velocity decrement and peak-to-peak, magnitude, IQR and mean of angular velocity were additionally only used in PD studies and are hypothesized to relate to the bradykinesia (slowing of movement) aspect in the (MDS-)UPDRS, emphasizing their clinical usefulness for early detection of bradykinesia symptoms (Garza-RodríguezSanchez-Fernandez et al., 2018). Coefficient of variation of both amplitude and velocity as well as rhythm, were included to reflect the interruptions as described in the (MDS-)UPDRS. CoV values are easy to calculate and interpret and showed to be sufficiently sensitive to discriminate between medication and stimulation states in PD patients from both finger-and wrist-worn sensors. This parameter could thus be implemented to evaluate objective intervention effects in large-scale medication or stimulation studies. In essential tremor and studies focusing on tremor in PD patients, occurrence and amplitude of peaks in specific frequency bands as well as power in these frequency bands were most often included, owing to the rhythmical aspect of tremor. However, the selected frequency bands were not always similar. The 4–12 Hz frequency band was most often considered as tremor (Heldman et al., 2011b; Ali et al., 2022), while Heo et al. and Kwon et al. used 3–12 Hz (Heo et al., 2015; Kwon et al., 2020), Patel et al. used a 3–8 Hz band and Schaefer et al. considered 7–12 Hz as the tremor frequency (Patel et al., 2009; Schaefer et al., 2021). Makabe et al. used a range of 8–12Hz and 20–25 Hz and Sprdlik et al. used the frequency distribution for highest maxima between 1 and 15 Hz (Makabe and Sakamoto, 2000; Šprdlík et al., 2011). Lopez-Bianco et al. used a high-pass filter with cut-off 4 Hz followed by low-pass filter with cut-off 8 Hz (López-Blanco et al., 2018). These differences suggest that a solid definition of tremor frequency is required in order to standardize instrumented tremor quantification, to allow comparison of methodologies on a large-scale cross-sectional level and to facilitate data merging and sharing.

In pathologies not related to PD or tremor, path length or similarity of hand trajectories were often calculated. This was the case in stroke, dyskinetic CP and pathologies associated with spasticity, reflecting the importance of the impact of the movement disorder on reaching movements. The frequent use of sensor features such as smoothness and jerk metrics might reflect the effect of the location of the brain lesions on the smooth execution of functional tasks and its impact on daily-life activities in these pathologies. For clinical implications, it is important to acknowledge the clinical differences between ‘rhythm’ and ‘jerk’. Rhythm is a self-constructed concept and its meaning is study-dependent, but the focus is on ‘regularly re-occurring events’ (Okuno et al., 2006; Martinez-Manzanera et al., 2016). Jerk-measures on the other hand are always based on the first derivative of the acceleration and/or the second derivative of the angular velocity signal and focus on the jerky, unpredictable movements in the signal. Rhythm thus implies stable and/or recurrent patterns in signals, whereas jerk measures represent quite the opposite. This is an important distinction that reflects the clinical difference between rhythmic movement disorders such as tremor and arrhythmic movement disorders such as dystonia and choreoathetosis (Sanger et al., 2010).

The clinical application of the included studies varied from discrimination of groups to prediction of severity levels and was closely related to the method used to obtain this specific result. With respect to the discrimination of groups, the sensor features sufficiently sensitive to detect differences between a control group and pathological patients could be used for early detection of e.g. PD or MS symptoms, allowing for early intervention and possibly preventing rapid worsening of symptoms. For the prediction of severity levels, all PD studies correlated the sensor features to the (MDS-)UPDRS, the AIMS or the Hoehn and Yahr scale. In CP and stroke, sensor features were correlated with the Melbourne Assessment Scale and ARAT, whereas in another CP study, the Jebsen-Taylor Test, the Quality of Upper Extremity Skills Test (QUEST) and the Box and Blocks Test were included. When the clinical application was the (side)effect of intervention, six out of 16 studies used sensor features to assess dyskinesia in PD patients, as this is a well-known levodopa-induced motor complication (Jankovic, 2005). The clinical scales in PD and tremor rate symptom severity, while the Melbourne Assessment Scale, the ARAT, the Jebsen-Taylor Test, the Quality of Upper Extremity Skills Test and the Box and Blocks Test in CP and stroke mainly evaluate upper extremity function. The severity of the movement disorder in stroke and CP is often dependent on the location of the brain lesion, which was not researched in detail in the included studies and has not been fully elucidated to date in most movement disorders (Bansil, 2012). To this end, wearable sensors provide opportunities for detailed exploration of the connection between the location of the brain lesion and the aetiology and severity of movement disorders.

IMUs have mostly been used to assess upper limb use and for detection of activity periods in daily life in patients with PD and/or essential tremor (Nguyen et al., 2017; Pham et al., 2017; Serrano et al., 2017), CP (Braito et al., 2018; Beani et al., 2019; Ahmadi et al., 2020) or stroke (Biswas et al., 2015), but their application to quantify movement disorders in the upper limb is less extensive. Activity measures mostly focus on the amount of time that acceleration measures exceed a pre-defined threshold (e.g., Activity Index), which yields information about the quantity of movement, but not about the quality. To facilitate follow-up of intervention or long-term rehabilitation programs, a combined assessment of both movement quantity and quality can provide more insights in both the presence and severity of movement disorders. Ideally, long-term monitoring is executed in a home-environment (i.e., low patient-burden while collecting long-term data), while a contact moment to record pathology-related tasks in a standardized setting could be added to the study protocol since this allows more specific data analysis, e.g., through the presence of video recordings of the performed tasks.

To maximise the use of wearable sensors for the quantification of upper limb movement disorders in clinical practice, one should acknowledge the differences in clinical symptoms between PD and tremor and movement disorders, such as dystonia and choreoathetosis. In PD, the expression of bradykinesia, hypokinesia and tremor is standardized and relatively easy recognisable. The features implemented should thus embody this pattern such as velocity decrement and mean/IQR of angular velocity for bradykinesia, movement decrement and range/RMS of angular displacement for hypokinesia and occurrence and amplitude of peaks in frequency bands for tremor. For dystonia and choreoathetosis, movement disorders known as being much less consistent, research should first focus on the search for sensor features capable of accurately discriminating between distinct movement disorders and their ability to quantify their severity. For this purpose, multi-centre studies are required, considering the low prevalence of individuals with dystonia and/or choreoathetosis.

For the quantification of spasticity, IMUs on the lower limb have been used to explore the relationship between maximal angular velocity and stretch velocity during passive stretches, but few studies focusing on upper limb measures with IMUs are available (Bar-On et al., 2014). When using IMUs for the instrumented assessment of spasticity, one should not only take into account active tasks but also passive fast and slow stretches to differentiate its neural and non-neural components as well as its velocity-dependent component. In the current review, only Bai et al. and Strohrmann et al. used IMUs to evaluate spasticity, and they included similar sensor features such as mean and standard deviation of acceleration and movement trajectory (Strohrmann et al., 2013; Bai et al., 2021). One extra challenge in spasticity may be range of motion restrictions, which can be evaluated using IMUs if sufficient passive movement is possible. If sensor placement on the hand or wrist is not possible due to severe positional deformities, the upper arm can be used as an alternative. Overall, IMUs have been scarcely used for the assessment of spasticity and the current review can serve as a facilitator to explore the different facets of spasticity using wearable sensors.

The use of these sensor features retrieved from one sensor on the hand, wrist or arm in combination with a home-based protocol to assess the effect of an intervention can greatly increase our understanding into the impact of current treatment management plans on the severity of upper limb movement disorders. The insights obtained for PD can accelerate the development of wearable sensors protocols in the remaining pathologies, provided that there is sufficient attention for the standardisation of protocols, tasks, feasibility and data analysis methods.

## Conclusion and future directions

Wearable sensors offer a myriad of opportunities for the quantification of movement disorders in multiple pathologies, but the abundance of available information could threaten its usability. Our findings illustrate that there are a lot of similarities between pathology-related sensor protocols and tasks, but the agreement is yet not sufficient to allow data pooling or international multi-centre studies. For this purpose, higher-level standardisation with respect to task selection and sensor feature extraction per pathology is strongly recommended. Although multiple sensors can provide a lot of information, researchers should think carefully about the balance between information gain and accessibility. One sensor on the index finger for PD or on the hand, wrist or forearm for other pathologies could be attached in a non-obstructive way, allowing for better adherence and less missing data due to e.g., battery loss. Current overview can support clinicians and researchers to select the most sensitive pathology-dependent sensor features and measurement methodologies for detection and quantification of upper limb movement disorders and for the objective evaluations of treatment effects.

## Data Availability

The original contributions presented in the study are included in the article/Supplementary Material, further inquiries can be directed to the corresponding author
