# Supplementary material for: Assessment of movement disorders using wearable sensors during upper limb tasks: A scoping review
Source: Front Robot AI. 2023 Jan 9;9:1068413. doi: 10.3389/frobt.2022.1068413 (PMC9879015; doi:10.3389/frobt.2022.1068413)
Supplement: Supplementary file 2 [file Table2.docx]

| Number of sensor(s) | Location of sensor(s) | Type | Goal population |
| --- | --- | --- | --- |
| 1 | Index finger tip | ACC | PD (Bravo et al., 2017; Makabe and Sakamoto, 2000; Okuno et al., 2006; Thanawattano et al., 2015) |
| 1 | Index finger tip | GYR | PD (Hoffman and McNames, 2011; Kim et al., 2011) |
| 1 | Index finger | Motion sensor | PD (Tamás et al., 2016) |
| 1 | Index finger | IMU | PD (Zhu and Miller, 2020), tremor (Heldman et al., 2011) |
| 1 | Dorsal hand | GYR | PD (Koop et al., 2006; Kwon et al., 2018) |
| 1 | Dorsal hand | Orientation sensor | Stroke (Kamper et al., 2002) |
| 1 | Dorsal hand | ACC | Tremor (Budini et al., 2014) |
| 1 | Wrist | ACC | PD (Griffiths et al., 2012; Habets et al., 2021; Schaefer et al., 2021), dystonia (Legros et al., 2004) , ataxia (Gupta et al., 2022), MS (Teufl et al., 2017) |
| 1 | Forearm | IMU | PD (Spasojević et al., 2017), stroke (Thies et al., 2009) |
| 1/2 (Ghassemi et al., 2016)† | Dorsal hand | IMU | PD (Bermeo et al., 2019; Bravo et al., 2016; Ghassemi et al., 2016; Rabelo et al., 2017), ataxia (Nguyen et al., 2020), tremor (McGurrin et al., 2021; Šprdlík et al., 2011) |
| 1/2 (Garza-Rodgrigues et al ; Oubre et al.; Strohrmann et al.,   Thomas et al.)† | Wrist(s) | IMU | PD (Garza-Rodríguez et al., 2018, 2020; Rigas et al., 2016; Thomas et al., 2018), stroke (Otten et al., 2015; Parnandi et al., 2010; Zhang et al., 2012), CP (Strohrmann et al., 2013), MS (Carpinella et al., 2014, 2015; Teufl et al., 2021), ataxia (Oubre et al., 2021), tremor (Gallego et al., 2012) |
| 1/2 (Jun et al.) † | Wrist(s) | GYR | PD (Jun et al., 2011; Salarian et al., 2007), tremor (López-Blanco et al., 2018) |
| 2 | Hand and wrist | IMU & ACC | PD (Shawen et al., 2020), ataxia (Tran et al., 2020) |
| 2 | Dorsal hand and index finger | IMU | PD (Martinez-Manzanera et al., 2018) |
| 2 | Wrist and index finger | ACC | PD (Martinez-Manzanera et al., 2016; Rahimi et al., 2015), tremor (Samotus et al., 2016) |
| 2 | Thumb and index finger | ACC | PD (Yokoe et al., 2009) |
| 2 | Thumb and index finger | GYR | PD (Lee et al., 2015; Summa et al., 2017) |
| 2 | Thumb and index finger | IMU | PD (Djurić-Jovičić et al., 2016; Espay et al., 2011; Heldman et al., 2011; Li et al., 2020; Liu et al., 2016; Park et al., 2021) |
| 3 | Index finger, forearm, upper arm | IMU | Ataxia (Dominguez-Vega et al., 2021; Martinez-Manzanera et al., 2018) |
| 3 | Index finger, hand, forearm | GYR | Tremor (Heo et al., 2015; Kwon et al., 2020) |
| 3 | Hand, forearm, upper arm | IMU | PD (Angeles et al., 2017; Chan et al., 2022), stroke (Knorr et al., 2005), tremor (Ali et al., 2022) |
| 3 | Wrists and sternum | ACC | HD (Bennasar et al., 2018) |
| 3 | Wrist, sternum and sacrum | IMU | Stroke (van Meulen et al., 2015) |
| 3 | Upper arms and trunk | IMU | CP (Newman et al., 2017) |
| 4 | Thumb, index finger, wrist, upper arm | IMU | Stroke (Del Din et al., 2011) |
| 4 | Fingers and wrist | IMU | PD (Cavallo et al., 2019) |
| 4 | Hands and forearms | IMU | PD (Lonini et al., 2018), tremor (Benito-León et al., 2019) |
| 4 | Wrists, trunk, head | IMU | PD (Romano et al., 2021) |
| 4 | Wrists and ankles | IMU | PD (Hssayeni et al., 2021; Pulliam et al., 2018), dyskinetic CP (den Hartog et al., 2022) |
| 4 | Wrist, sternum, thigh, foot | IMU | PD (Zwartjes et al., 2010) |
| 4 | Hand, forearm, upper arm, shoulder | IMU | Spasticity (Bai et al., 2021) |
| 5 | Thumb, index finger, hand, wrist, upper arm | IMU | PD (di Biase et al., 2018) |
| 5 | Hand, wrist, upper arm, shoulder, sternum | IMU | MS (Ketteringham et al., 2011; Western et al., 2019) |
| 5 | Hand, forearm, upper arm, head, back | Orientation sensors | Dyskinetic CP (Sanger, 2006) |
| 6 | Thumb, index finger, hand, forearm, upper arm, sternum | ACC | Stroke (Hester et al., 2006; Patel et al., 2010) |
| 6 | Hands, forearms, upper arms | IMU | PD (Lukšys et al., 2018), tremor (Chan et al., 2018) |
| 6* | Dorsal hands, wrists, ankles | IMU | Ataxia (Krishna et al., 2019) |
| 6 | Hand, scapula, thorax, sacrum, posterior of the head, lateral shank | Magnetic motion tracker | PD (Chelaru et al., 2010) |
| 6 | Wrists, waist, chest, ankles | IMU | PD (Tsipouras et al., 2012) |
| 7 | Hands, wrists, upper arms, sternum | IMU | Stroke (Repnik et al., 2018) |
| 7 | Wrists, upper arms, trunk, upper legs | ACC | PD (Hoff et al., 2001; Keijsers et al., 2003) |
| 8 | Hands, wrists, upper arms, shoulder | IMU | Stroke (Delrobaei et al., 2016) |
| 8 | Forearms, upper arms, sternum, thighs, right shin | ACC | PD (Bonato et al., 2004) |
| 8 | Forearms, upper arms, shins, upper legs | ACC | PD (Cole et al., 2010; Patel et al., 2009) |
| 8 | index finger, dorsal hand, wrist, dorsum foot, sternum, upper-back, ankles | IMU | Ataxia (Kashyap et al., 2020) |
| 11 | Dorsal hand and fingers | IMU | PD (van den Noort et al., 2017) |
| 17 | Hands, wrists, upper arms, clavicle’s, sternum, head, pelvis, legs, feet | IMU | PD (Delrobaei et al., 2018) |
| Unknown | Upper limb (no specification) | ACC | CP (Kim et al., 2018) |

*Table S3: Number, type and location of included sensors per reference. ACC = accelerometer; GYR = gyroscope; IMU = inertial measurement unit. † Either 1 sensor was placed on the dorsal hand, or 1 on each hand*. ** Krishna et al. used one sensor but placed it subsequently on the L/R wrist, L/R dorsal hand and L/R ankle.*
