# Supplementary material for: Assessment of movement disorders using wearable sensors during upper limb tasks: A scoping review
Source: Front Robot AI. 2023 Jan 9;9:1068413. doi: 10.3389/frobt.2022.1068413 (PMC9879015; doi:10.3389/frobt.2022.1068413)
Supplement: Supplementary file 4 [file Table3.docx]

Table S3: Sensor features classified by pathology. STD = standard deviation; RMS = root-mean-square; MAX = maximal; VAR = variance; IQR = inter-quartile range.

| PD | Stroke | CP | Ataxia | HD | Tremor | MS | Spasticity | Dystonia |
| --- | --- | --- | --- | --- | --- | --- | --- | --- |
| - Execution time  - Movement frequency  - Mean acceleration/angular velocity  - STD, RMS, MAX, VAR acceleration/angular   velocity  - Timing MAX acceleration/angular velocity  - Mean, median, RMS, STD, MAX, range, IQR   acceleration  - Mean, median, RMS, STD, range, IQR angular   velocity  - Max linear velocity  - RMS, range, IQR angular displacement  - Mean amplitude  - Range of jerk & angular acceleration  - Peak-to-peak, magnitude angular velocity  - Segment velocity  - Kurtosis  - Skewness  - Sample entropy  - Approximate, Shannon entropy  - Gini index  - RMS jerk  - Jerk metric  - Coefficient of variation  - Rhythm  - Correlation between axes  - Peak of normalized cross-correlation   from pairs of acceleration time series  - Lag of first peak in autocorrelation acceleration  - Bradykinesia Index  - Movement, velocity decrement  - Amplitude of modulation acceleration  - Normalized mean squared error between a   target signal and its forward linear prediction  - Dominant frequency component  - Second dominant frequency  - Energy & angular velocity  - Amplitude and dominant frequency of   modulation associated with acceleration  - Fractal dimension acceleration  - Spectral, peak, second peak, total, mean power  - Spectral entropy  - Spectral Arc Length | - Execution time  - Mean, VAR acceleration   & angular velocity - Mean, RMS acceleration  - Max linear velocity - Mean amplitude - Range of jerk & angular   acceleration  - Rotational jerk index  - Sample, approximate   entropy  - RMS jerk  - Jerk metric  - Correlation between axes  - Variability  - Path length  - Similarity of hand   trajectories  - Dominant frequency   component  - Dominant frequency of   jerk  - Energy & angular   velocity  - Spectral power  - Spectral entropy | - Execution time - Mean, MAX, RMS   acceleration &   angular velocity - STD acceleration - Range angular   velocity  - Shannon entropy - Jerk metric - Higuchu’s fractal   dimension  - Path length  - Elevation angle - Band power - Spectral Arc   Length | - Execution time - Mean, MRS, VAR   acceleration &  angular velocity  - STD, MAX   acceleration  - STD, median,   range angular   velocity  - Kurtosis  - Sample Entropy  - Fuzzy entropy  - Gini index  - Path length  - Dominant   frequency of jerk  - Resonant   frequency  - Peak power  - Spectral entropy | - Mean, STD   acceleration  - Max, STD, RMS &   min/max peak height  - Sample, permutation   entropy  - Lyapunov exponent  - Recurrence rate &   determinism  - Average diagonal   line  - Correlation   between axes  - Energy acceleration  - Average magnitude   of 1^st^ five STFT   components  - Component   entropy | - Execution time  - Mean, STD   acceleration  - RMS angular   velocity  - RMS angular   displacement/   movement   amplitude  - Coefficient of   variation  - Spectral, peak   power  - Specific tremor   index  - Tremor   frequency/   amplitude | **-** Execution time  **-** Mean angular   displacement  **-** Jerk metric  **-** Peak power  **-** Specific tremor   index | - Jerk metric  - Smoothness  - Path length | - Spectral   power |
